# Next-generation LMP2A-targeting TCR-recombinant T cells with inducible IL-18 expression to treat EBV-associated malignancies

**DOI:** 10.1016/j.omton.2026.201265

**Published:** 2026-06-11

**Authors:** Agnes Bonifacius, Philip Mausberg, Friederike Floegel, Anna Christina Dragon, Sabine Tischer-Zimmermann, Sven Stoll, Pegah Rahmati, Peter Spieler, María Fernanda Lammoglia Cobo, Anne Halenius, Hinrich Abken, Rainer Blasczyk, Thomas Nerreter, Michael Hudecek, Axel Schambach, Leo Hansmann, Britta Maecker-Kolhoff, Britta Eiz-Vesper

**Affiliations:** 1 Institute of Transfusion Medicine and Transplant Engineering, Hannover Medical School, 30625 Hannover, Germany; 2 nextGENERATION Medical Scientist Program, Dean’s Office for Academic Career Development, Hannover Medical School, 30625 Hannover, Germany; 3 German Center for Infection Research (DZIF), Thematical Translation Unit-Immunocompromised Host (TTU-IICH), Partner Site Hannover-Braunschweig, Hannover, Germany; 4 Chair for Cellular Immunotherapy, Department of Medicine II, University Hospital Würzburg, 97080 Würzburg, Germany; 5 Department of Hematology, Oncology and Tumor Immunology, Charité-Universitätsmedizin Berlin, Corporate Member of Freie Universität Berlin and Humboldt-Universität zu Berlin, 10117 Berlin, Germany; 6 Institute of Virology, Medical Center University of Freiburg, 79106 Freiburg, Germany; 7 Department Genetic Immunotherapy, Leibniz Institute for Immunotherapy, 93053 Regensburg, Germany; 8 Institute of Experimental Hematology, Hannover Medical School, 30625 Hannover, Germany; 9 Department of Internal Medicine III, Universitätsklinikum Regensburg, 93053 Regensburg, Germany; 10 Department of Pediatric Hematology and Oncology, Hannover Medical School, 30625 Hannover, Germany; 11 Fraunhofer Institute for Cell Therapy and Immunology (IZI), Leipzig & Branch Site Cellular Immunotherapy, 97070 Würzburg, Germany; 12 National Center for Tumor Diseases (NCT), Würzburg, Germany; 13 Bavarian Cancer Research Center (BZKF), Würzburg, Germany

**Keywords:** adaptive immunity, Epstein-Barr virus, post-transplant lymphoproliferative disorder, immunotherapy, T cells, TCR engineering, 4th generation CAR, T-cell therapy, LMP2A

## Abstract

Epstein-Barr virus (EBV) infects more than 90% of the population and establishes a lifelong persistence in memory B cells, passing through several latency stages (I–III). In immunocompromised patients, EBV infections and reactivations can lead to severe complications, such as post-transplant lymphoproliferative disorder (PTLD), a malignant B cell lymphoproliferation. The EBV latent membrane protein 2A (LMP2A) induces activation and proliferation of infected B cells and is expressed in latency stages II/III, that are associated with several EBV malignancies. Here, T cell receptor (TCR)-engineered T cells based on a TCR recognizing the clinically relevant HLA-A∗02:01-restricted LMP2A-derived peptide CLGGLLTMV (A∗02_LMP2A_CLG_) and equipped with a TCR-inducible cassette for IL-18 release (iIL-18_LMP2A_TCR-T cells) aiming to prevent exhaustion and promote remodeling of the immunosuppressive tumor microenvironment (TME) were developed. The iIL-18_LMP2A_TCR-T cells exhibited improved cytotoxicity against HLA-A∗02:01^+^ EBV-infected B-lymphoblastoid cell lines (EBV^+^ B-LCL^A∗02:01^) serving as *in vitro* PTLD model, when compared to LMP2A_TCR-T cells without iIL-18. The superior functionality of iIL-18_LMP2A_TCR-T cells was further confirmed in multicellular tumor spheroid (MCTS) models, where they mediated sustained control of EBV^+^ B-LCL^A∗02:01^ growth, highlighting their potential as an effective therapeutic approach for the immune-mediated eradication of EBV-associated malignancies, including PTLD.

## Graphical abstract

**Figure:**
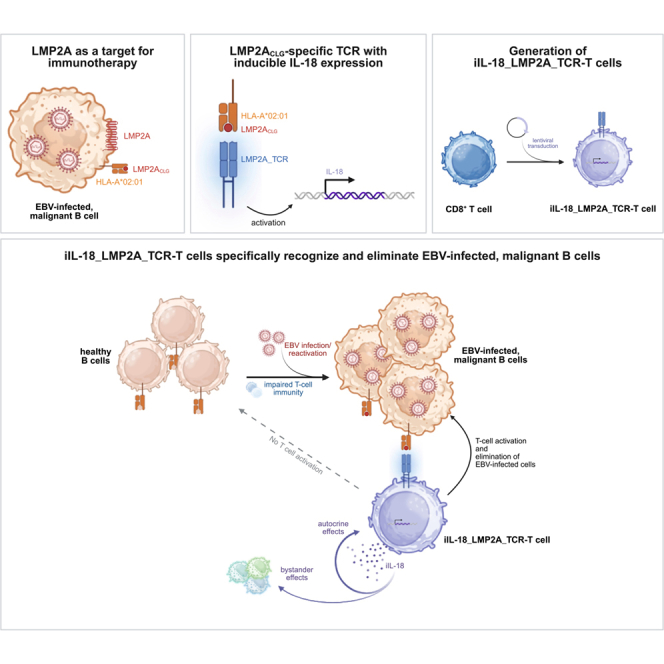

## Teaser

Eiz-Vesper and colleagues developed TCR-engineered T cells with inducible IL-18 production that target Epstein-Barr virus (EBV) latency protein LMP2A. The iIL-18-mediated improved T cell functionality as well as superior recruitment and activation of bystander immune cells suggests enhanced anti-tumor responses in EBV-associated malignancies, offering a potent approach for targeted immunotherapy.

## Introduction

During primary infection, Epstein-Barr virus (EBV) establishes latency in B cells and oral epithelial cells and remains in memory B cells life-long. It passes through different latency stages (I–III), which are characterized by differential expression of EBV-associated genes, such as Epstein-Barr nuclear protein (EBNA)1-3 and latent membrane protein (LMP) 1, 2A, and 2B.^1^^,^^2^ While in healthy individuals, EBV-infected B cells are efficiently controlled by EBV-specific T cells, in immunocompromised individuals, such as patients following hematopoietic stem cell transplantation (HSCT) or solid organ transplantation (SOT), uncontrolled proliferation of EBV-infected B cells can lead to various malignancies.^3^^,^^4^ The majority of EBV^+^ malignancies are primarily associated with latency stages II or III; amongst them are B-lymphoproliferative diseases, including post-transplant lymphoproliferative disorder (PTLD).^5^^,^^6^

Besides reduction of immunosuppression, the main therapeutic option is the administration of rituximab, a monoclonal antibody targeting CD20 on B cells, with or without chemotherapy.^7^^,^^8^ Despite response rates of 60%–80%, this approach is associated with increased susceptibility to opportunistic infections due to hypogammaglobinemia and severe side effects of chemotherapy. Treatment options for relapsed/refractory PTLD are limited by development of resistances to rituximab.^9^^,^^10^^,^^11^ In CD30^+^ PTLDs (70%–85% of the cases), treatment with the CD30-targeting monoclonal antibody-drug conjugate brentuximab vedotin can be considered.^12^^,^^13^

Alternatively, adoptive T cell therapy with EBV-specific T cells (EBV-VSTs), generated from stem cell, related or unrelated third-party donors, has emerged as an attractive therapeutic option to restore the functional antiviral immunity.^9^^,^^14^^,^^15^^,^^16^^,^^17^^,^^18^^,^^19^ Despite overall good tolerability and response rates between 50% and 70% in stem cell and solid organ transplanted patients, successful transfer of EBV-VSTs depends on the availability of donors with (at least partial) human leukocyte antigen (HLA) match to the recipient and sufficient EBV-specific memory T cell frequencies. In addition, the efficacy of antiviral T cells is still limited by the tumor microenvironment (TME), which includes the dense extracellular matrix, irregular vasculature, and immunosuppressive cells that hinder T cell migration and suppress antitumor function.^20^^,^^21^^,^^22^

New treatment options, such as the administration of chimeric antigen receptor (CAR)-T cells targeting the B cell antigen CD19 for relapsed/refractory PTLD after SOT are under evaluation. A retrospective analysis of SOT-related PTLD showed an overall response rate of 64% (55% complete remission).^23^ However, three patients (14%) experienced graft rejection after CAR-T therapy. Furthermore, remaining challenges such as the inability to differentiate between EBV-infected and uninfected B cells and the associated increased infection susceptibility are yet unsolved.^24^ CARs specifically targeting EBV-infected cells via binding to EBV gp350 have been described; however, these failed to control lymphoma development *in vivo*, likely due to low antigen expression and antigen loss.^25^ In an attempt to improve the activity and functionality of T cells within the TME, CAR-T cells secreting immune-activating cytokines such as interleukin (IL-)12 or IL-18, have been developed and shown to mediate enhanced anti-tumor responses due to autocrine effects as well as recruitment of bystander immune cells.^26^ This approach was implemented when using a T cell receptor (TCR)-like antibody recognizing an EBNA3C-derived peptide in context of HLA-B∗35 as antigen binding domain; here the equipment with an inducible IL-12 (iIL-12) cassette resulted in improved effector functionality.^27^

Overall, selectively targeting viral proteins presents a promising strategy for adoptive T cell therapies to treat EBV-induced malignancies. Despite the development of EBV-specific CARs, these face challenges due to in part low antigen expression. Since TCRs have a higher antigen sensitivity compared to CARs, T cells equipped with recombinant TCRs have the potential to overcome these limitations of CAR-T cells. LMP2A is a key protein found in the majority of EBV-associated malignancies (latency stages II-III), such as PTLD, and was identified as the third priority cancer antigen in a National Cancer Institute (NCI) pilot project.^28^^,^^29^ The LMP2A-derived HLA-A∗02:01-restricted nonamer CLGGLLTMV (CLG, aa 426–434) was identified as one of the most immunogenic peptides of EBV.^30^ Together with the high prevalence of HLA-A∗02:01, ranging between 24% and 30% in the European population, A∗02_LMP2A_CLG_ represents a clinically relevant target for immunotherapy.^31^

Here, we developed TCR-engineered T cells recognizing the A∗02_LMP2A_CLG_ epitope (LMP2A_TCR-T cells). The TCR was recently identified in healthy donor T cells, expanded in the presence of the A∗02_LMP2A_CLG_ epitope, and reported to trigger IFN-γ production when recognizing EBV^+^ B-LCLs.^32^ LMP2A_TCR-T cells were further equipped with inducible IL-18 (iIL-18) release (iIL-18_LMP2A_TCR-T cells) hypothesizing that iIL-18 will considerably improve their functionality. Generated iIL-18_LMP2A_TCR-T cells were evaluated for their cytotoxic capacity toward EBV^+^ B-LCLs in comparison to LMP2A_TCR-T cells in two-dimensional (2D) as well as multicellular tumor spheroids (MCTS) 3D models. Overall, we demonstrated that iIL-18_LMP2A_TCR-T cells are a promising strategy for the treatment of EBV-related malignancies and superior to LMP2A_TCR-T cells due to their improved functionality and potential to recruit and activate bystander immune cells.

## Results

### (iIL-18_)LMP2A_TCRs recognize the A∗02_LMP2A_CLG_ epitope and mediate specific elimination of A∗02_LMP2A_CLG_-expressing cells

To confirm the specificity of the generated TCR constructs (Figure S1A), namely iIL-18_LMP2A_TCR and LMP2A_TCR (from here on referred to as (iIL-18_)LMP2A_TCRs when both are addressed), toward the A∗02_LMP2A_CLG_ epitope, they were transduced into a Jurkat JE6-1-derived nuclear factor kappa-light-chain-enhancer (NF-κB) reporter T cell line. These were then co-cultured with the EBV^−^ SPI-801 cell line engineered to express HLA-A∗02:01 (SPI-801^A^^∗^^02:01^; Figure S1B) and loaded with the LMP2A-derived CLG peptide (SPI-801^A∗^^02:01^_CLG_). Induction of NF-κB signaling was observed in co-cultures of (iIL-18_)LMP2A_TCR-expressing JE6-1 cells with SPI-801^A∗^^02:01^_CLG_ but not SPI-801^A∗^^02:01^, and the induction of NF-κB activation was increased in a CLG concentration-dependent manner (Figures S1C and S1D), indicating antigen-dependent activation through the recombinant TCR.

Primary CD8^+^ T cells from healthy individuals were transduced to express (iIL-18_)LMP2A_TCR constructs and expanded for 12–15 days (Figure S2A). (iIL-18_)LMP2A_TCR-T cells mainly consisted of central memory (TCM) and effector memory (TEM) T cells, with no significant differences between LMP2A_TCR- and iIL-18_LMP2A_TCR-T cells (Figure S2B). Moreover, the frequency of transgenic TCR-expressing cells was comparable between LMP2A_TCR- and iIL-18_LMP2A_TCR-T cells (Figure S2C). Baseline exhaustion (LAG3 and TIM3) and activation (CD69, CD137, and CD25) levels during generation were found to be comparable between both (iIL-18_)LMP2A_TCR-T cells and untransduced CD8^+^ T cells (Figure S2D). Of note, expression of CD25 and TIM3 increased early during expansion and remained at this level with no differences observed between (iIL-18_)LMP2A_TCR-T cells and untransduced CD8^+^ T cells. Exposure of SPI-801^A∗^^02:01^_CLG_ to LMP2A_TCR- or iIL-18_LMP2A_TCR-T cells resulted in significantly reduced viability of SPI-801^A∗^^02:01^_CLG_ (mean 9.31% and 11.71%, respectively) but not SPI-801^A∗^^02:01^ target cells (mean 48.61% and 44.31%, respectively) compared to target cells cultured alone (SPI-801^A∗^^02:01^: 60.84%, SPI-801^A∗^^02:01^_CLG_: 63.16% viable cells) (Figure S2E). No differences between LMP2A_TCR- and iIL-18_LMP2A_TCR-T cells were observed.

In summary, both (iIL-18_)LMP2A_TCRs specifically recognize the HLA-A∗02:01_CLG epitope, which resulted in potent cytotoxicity of (iIL-18_)LMP2A_TCR-T cells toward cells expressing the A∗02_LMP2A_CLG_ epitope.

### (iIL-18)_LMP2A_TCR-T cells recognize HLA-A∗02^+^ EBV-infected B-LCLs serving as *in vitro* PTLD model

**Figure 1:**
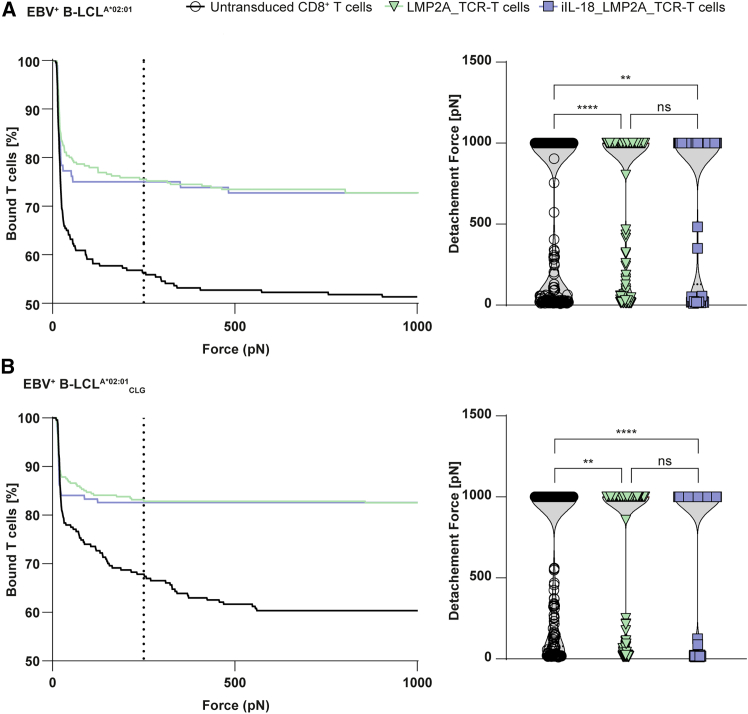
(iIL-18_)LMP2A_TCR-T cells show high avidity toward EBV^+^ B-LCL^A^^∗^^02:01^_(CLG)_ The avidity of (iIL-18_)LMP2A_TCR-T cells to autologous EBV^+^ B-LCL^A^^∗^^02:01^_(CLG)_ was evaluated using acoustic force microfluidicmicroscopy (z-Movi). For that, (iIL-18_)LMP2A_TCR-T cells were generated from human primary CD8^+^ T cells via lentiviral transduction. Untransduced CD8^+^ T cells served as negative control. EBV^+^ B-LCL^A^^∗^^02:01^ were generated using PBMCs isolated from HLA-A∗02:01^+^ healthy donors. EBV^+^ B-LCL^A^^∗^^02:01^_CLG_ were prepared by overnight loading with the HLA-A∗02:01-restricted LMP2A-derived peptide CLGGLLTMV (CLG). Data are shown as representative immune synapse-binding avidity and violin detachment force plots of untransduced CD8^+^ T cells, LMP2A_TCR-T cells or iIL-18_LMP2A_TCR-T cells. (A) EBV^+^ B-LCL^A∗02:01^, (B) EBV^+^ B-LCL^A^^∗^^02:01^_CLG_. Data represent experiments from one microfluidic chip. Statistical analysis was performed using Kruskal-Wallis test and uncorrected Dunn’s test. ns: not significant; ∗∗*p* ≤ 0.01; ∗∗∗∗*p* ≤ 0.0001.

EBV-infected B-LCLs serving as well-established *in vitro* PTLD model were used as target cells. To confirm binding of the LMP2A_TCR to the A∗02_LMP2A_CLG_ epitope on EBV^+^ B-LCLs generated from an HLA-A∗02:01-positive donor (EBV^+^ B-LCL^A^^∗^^02:01^, Figure S3), z-Movi analysis was performed (Figure 1). While untransduced CD8^+^ T cells poorly bound to EBV^+^ B-LCL^A^^∗^^02:01^, both (iIL-18_)LMP2A_TCR-T cells bound EBV^+^ B-LCL^A^^∗^^02:01^ with high avidity (Figure 1A). In that, the interaction formed rapidly after 5 min of incubation and approximately 75% of both (iIL-18_)LMP2A_TCR-T cells were still attached to the monolayer of EBV^+^ B-LCL^A^^∗^^02:01^ when subjected to an acoustic force higher than 250 piconewton (pN), while at the same acoustic force only 56.3% of untransduced CD8^+^ T cells remained bound. Statistical analysis of individual detachments in relation to the applied force revealed significantly higher avidity of (iIL-18_)LMP2A_TCR-T cells when compared to untransduced CD8^+^ T cells. To increase target availability by saturation of the A∗02_LMP2A_CLG_ epitope, a similar experiment was performed using EBV^+^ B-LCL^A^^∗^^02:01^ exogenously loaded with the CLG peptide (B-LCL^A^^∗^^02:01^_CLG_), resulting in an even increased avidity of (iIL-18_)LMP2A_TCR-T cells (Figure 1B).

In summary, (iIL-18_)LMP2A_TCR-T cells specifically recognize and bind to EBV^+^ B-LCL^A^^∗^^02:01^ endogenously processing intracellular EBV-derived proteins and presenting the A∗02_LMP2A_CLG_ epitope.

### Superior cytotoxic capacity of iIL-18_LMP2A_TCR-T cells toward EBV^+^ B-LCLs compared to LMP2A_TCR-T cells

**Figure 2:**
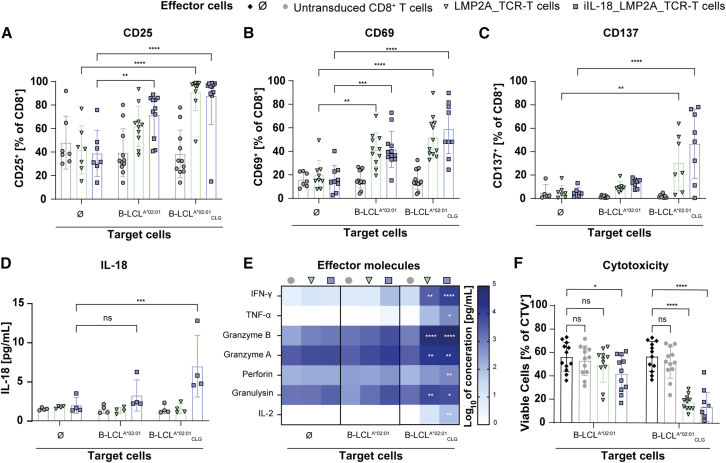
(iIL-18_)LMP2A_TCR-T cells are specifically activated, release proinflammatory cytokines and mediate specific cytotoxicity toward EBV^+^ B-LCL^A^^∗^^02:01^_(CLG)_ The specific recognition and elimination of EBV^+^ B-LCL^A^^∗^^02:01^_(CLG)_ by autologous or allogeneic (iIL-18_)LMP2A_TCR-T cells was evaluated using multicolor flow cytometry. For that, (iIL-18_)LMP2A_TCR-T cells were generated from human primary CD8^+^ T cells via lentiviral transduction. Untransduced CD8^+^ T cells served as negative control. EBV^+^ B-LCL^A^^∗^^02:01^ were generated using PBMCs isolated from HLA-A∗02:01^+^ healthy donors. EBV^+^ B-LCL^A^^∗^^02:01^_CLG_ were prepared by overnight loading with the HLA-A∗02:01-restricted LMP2A-derived peptide CLGGLLTMV (CLG). (iIL-18_)LMP2A_TCR-T cells were co-cultured with CellTrace Violet (CTV)-labeled EBV^+^ B-LCL^A^^∗^^02:01^_CLG_ in an E:T ratio of 1:1 for 48 h. T cells cultured alone and target cells cultured in absence of T cells served as control for baseline activation and target cell viability, respectively. (A) Frequencies of CD25^+^ cells (B) CD69^+^ cells, and (C) CD137^+^ cells among CD8^+^ T cells determined via flow cytometry. (D) IL-18 concentration in the cell culture supernatant was determined via LEGENDplex (*n* = 4). (E) The heatmap shows the concentration of indicated analytes in cell culture supernatants measured via LEGENDplex (*n* = 4). (F) Graph shows the frequencies of viable target cells (7-AAD^-^ among CTV^+^ cells) determined via flow cytometry (*n* = 12). Data are presented as (A–D, and F) mean ± SD with each symbol representing data from one independent donor or as (E) mean. Statistical analysis was performed using two-way ANOVA and Dunnett’s multiple comparisons test/Tukey’s multiple comparisons test and compared to T cells only (A–E) or target cells only (F). ∗*p* ≤ 0.05; ∗∗*p* ≤ 0.01; ∗∗∗∗*p* ≤ 0.0001.

Following the confirmed binding of (iIL-18_)LMP2A_TCR-T cells to EBV^+^ B-LCL^A^^∗^^02:01^_(CLG)_, their activation and cytotoxic capacity were evaluated by flow cytometry and multiplex analysis (Figure 2). Expression of CD25 and CD69 was significantly upregulated, while expression of CD137 was slightly upregulated on iIL-18_LMP2A_TCR-T cells upon recognition of EBV^+^ B-LCL^A^^∗^^02:01^ after 48 h, compared to T cells cultured alone (CD25: 71.52% vs. 38.91%, CD69: 41.65% vs. 16.70%, CD137: 13.08% vs. 5.28%) (Figures 2A–2C). Activation of LMP2A_TCR-T cells in response to EBV^+^ B-LCL^A^^∗^^02:01^ occurred with a similar trend and to the same extent for CD69 (41.47% vs. 19.75%) but was lower for CD25 (61.89% vs. 41.84%) and CD137 (9.90% vs. 5.58%) when compared to iIL-18_LMP2A_TCR-T cells. The activation of both (iIL-18_)LMP2A_TCR-T cells significantly increased upon recognition of EBV^+^ B-LCL^A^^∗^^02:01^_CLG_, with higher frequencies of CD137^+^ cells in iIL-18_LMP2A_TCR-T cells (46.89%) compared to LMP2A_TCR-T cells (30.47%). Overall, activation of iIL-18_LMP2A_TCR-T cells upon target recognition was slightly higher compared to LMP2A_TCR-T cells, although these differences did not reach statistical significance. The specific release of IL-18 by iIL-18_LMP2A_TCR-T cells upon target cell recognition was confirmed by the detection of IL-18 in cell culture supernatants, whereas IL-18 concentrations remained at background level in cell culture supernatants of LMP2A_TCR-T cells (Figure 2D). IL-18 concentrations were increased by 1.9- and 4.2-fold upon co-culture of iIL-18_LMP2A_TCR-T cells with EBV^+^ B-LCL^A^^∗^^02:01^ and EBV^+^ B-LCL^A^^∗^^02:01^_CLG_, respectively.

To understand how the release of iIL-18 influences the inflammatory profile of iIL-18_LMP2A_TCR-T cells, the secretion of key effector molecules was analyzed (Figure 2E). While the cytokine release profile suggested a trend toward superior secretory capacity of iIL-18_LMP2A_TCR-T cells compared to LMP2A_TCR-T cells, these differences were not statistically significant.

The recognition of EBV^+^ B-LCL^A^^∗^^02:01^_CLG_ by (iIL-18_)LMP2A_TCR-T cells resulted in significantly increased production of effector molecules IFN-γ, granzyme B, granzyme A, and granulysin. Of note, significantly increased concentrations of TNF-α (fold-increase 2,221), perforin (fold-increase 3.52), and IL-2 (fold-increase 1,077) were detected in co-cultures of iIL-18_LMP2A_TCR-T cells upon recognition of EBV^+^ B-LCL^A^^∗^^02:01^_CLG_, which was not the case for LMP2A_TCR-T cells. In line with slightly enhanced activation, overall higher levels of effector molecules were secreted by iIL-18_LMP2A_TCR-T cells compared to LMP2A_TCR-T cells. Although no significant upregulation of effector molecules in supernatants of iIL-18_LMP2A_TCR-T cells co-cultured with EBV^+^ B-LCL^A^^∗^^02:01^ was observed, data indicate a trend toward increased production.

To determine whether activation of the inducible cassette had an influence on the cytotoxic activity of iIL-18_LMP2A_TCR-T cells, specific tumor lysis was assessed (Figure 2F). Importantly, the analysis of cytotoxic properties toward EBV^+^ B-LCL^A^^∗^^02:01^ revealed that iIL-18_LMP2A_TCR-T cells, but not LMP2A_TCR-T cells, were able to significantly reduce viability of EBV^+^ B-LCL^A^^∗^^02:01^ (mean 42.2% and 49.6%, respectively) when compared to target cells cultured alone (mean 56.2%). In contrast, viability of EBV^+^ B-LCL^A^^∗^^02:01^_CLG_ (mean 57.0%) was significantly reduced by both iIL-18_LMP2A_TCR-T cells (mean 13.9%) and LMP2A_TCR-T cells (mean 17.7%), with no impact of iIL-18. No effects of untransduced CD8^+^ T cells that might be attributed to alloreactivity or presence of endogenous EBV-specific T cells were observed.

In summary, these findings demonstrate the successful and specific activation of the inducible cytokine cassette in iIL-18_LMP2A_TCR-T cells upon recognition of the HLA-A∗02:01_CLG_ epitope, resulting in slightly enhanced cytotoxicity of iIL-18_LMP2A_TCR-T cells compared to LMP2A_TCR-T cells.

### Live-cell imaging highlights superior cytotoxic potential of iIL-18_LMP2A_TCR-T cells

**Figure 3:**
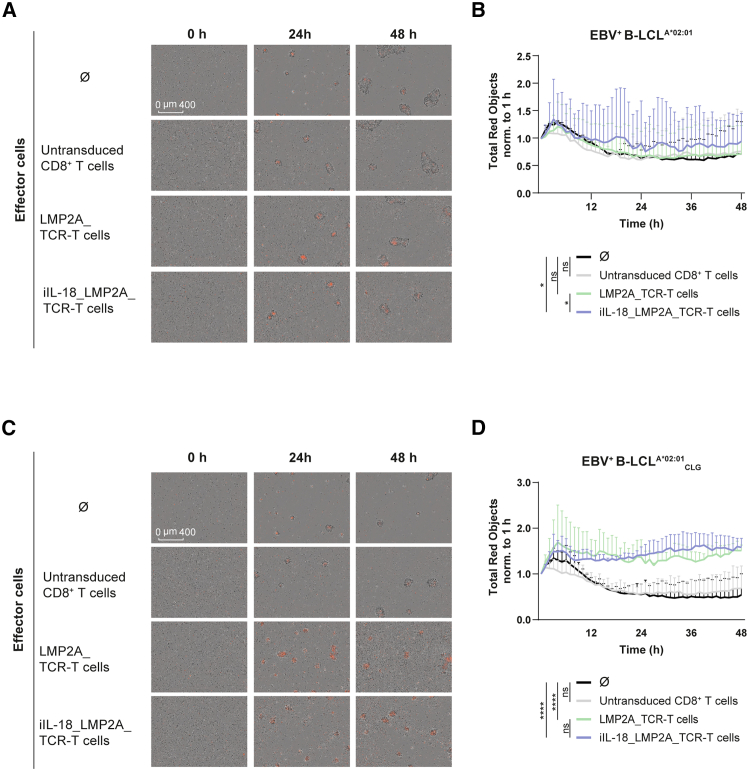
(iIL-18)_LMP2A_TCR-T cells mediate elimination of B-LCL^A^^∗^^02:01^ The specific recognition and elimination of autologous or allogeneic EBV^+^ B-LCL^A^^∗^^02:01^ by (iIL-18_)LMP2A_TCR-T cells was evaluated using live cell imaging. For that, (iIL-18_)LMP2A_TCR-T cells were generated from human primary CD8^+^ T cells via lentiviral transduction. Untransduced CD8^+^ T cells served as negative control. EBV^+^ B-LCL^A^^∗^^02:01^ were generated using PBMCs isolated from HLA-A∗02:01^+^ healthy donors and EBV^+^ B-LCL^A^^∗^^02:01^_CLG_ were prepared by overnight loading with CLG. (iIL-18_)LMP2A_TCR-T cells were co-cultured with EBV^+^ B-LCL^A^^∗^^02:01^_CLG_ in an E:T ratio of 1:1 for 48 h. Target cells cultured in absence of T cells served as control for baseline viability. Propidium iodide (PI) was used to assess cell death using the IncuCyte SX1 and a 10× short-distance objective lense. Scale bars in images represent 400 μm. (A) Representative microscopic overlayed phase-contrast and red fluorescence images of EBV^+^ B-LCL^A^^∗^^02:01^ cultured in presence of effector cells at indicated time points. (B) Quantitative analysis of the PI signal as total red objects normalized to the scan at 1 h, shown as mean + SD (*n* = 3). (C) Representative microscopic overlayed phase-contrast and red fluorescence images of EBV^+^ B-LCL^A^^∗^^02:01^_CLG_ cultured in presence of effector cells at indicated time points. (D) Quantitative analysis of the PI signal as total red objects normalized to the scan at 1 h, shown mean + SD (*n* = 3). Statistical analysis was performed using two-way ANOVA and Tukey’s multiple comparisons test. Results are shown for comparisons to target cells only and comparison between LMP2A_TCR-T cells and iIL-18_LMP2A_TCR-T cells. ns not significant; ∗*p* ≤ 0.05; ∗∗∗∗*p* ≤ 0.0001.

To follow the cytotoxic potential of both (iIL-18_)LMP2A_TCR-T cells toward EBV^+^ B-LCL^A^^∗^^02:01^_(CLG)_ in real-time, we employed live-cell imaging using propidium iodide (PI) to monitor target cell death (Figure 3). PI-mediated red fluorescence was low in EBV^+^ B-LCL^A^^∗^^02:01^ cultured alone or in presence of untransduced CD8^+^ T cells over the entire period of incubation time (Figure 3A). In contrast, a significantly higher PI signal was observed over time in co-cultures of EBV^+^ B-LCL^A^^∗^^02:01^ with iIL-18_LMP2A_TCR-T cells but not LMP2A_TCR-T cells, confirming the cytotoxic potential of iIL-18_LMP2A_TCR-T cells toward EBV^+^ B-LCL^A^^∗^^02:01^ (Figure 3B). Importantly, the cytotoxic capacity of iIL-18_LMP2A_TCR-T cells was significantly higher compared to LMP2A_TCR-T cells. Alongside previous results, a significant cytotoxic potential of both (iIL-18_)LMP2A_TCR-T cells toward EBV^+^ B-LCL^A^^∗^^02:01^_CLG_ was observed, and in this setting no significant difference between LMP2A_TCR-T cells and iIL-18_LMP2A_TCR-T cells was observed (Figures 3C and 3D).

Taken together, these results emphasize the significantly superior and sustained cytotoxic capacity of iIL-18_LMP2A_TCR-T cells toward EBV^+^ B-LCL^A^^∗^^02:01^ compared to LMP2A_TCR-T cells.

### Recognition of B-LCL^A^^∗^^02:01^_CLG_ by iIL-18_LMP2A_TCR-T cells induces activation and recruitment of bystander cells

**Figure 4:**
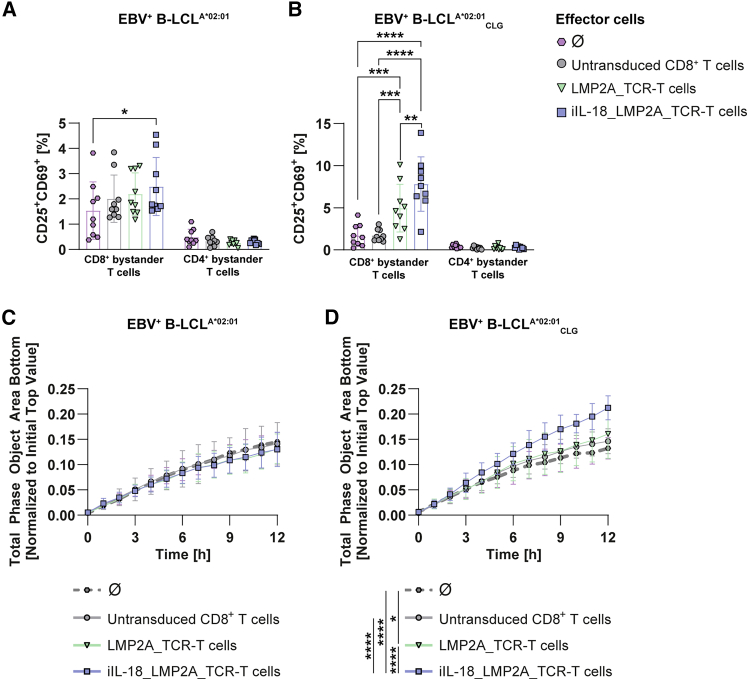
(iIL-18)_LMP2A_TCR-T cells induce bystander CD8^+^ T cell activation and recruitment of innate immune cells upon recognition of B-LCL^A^^∗^^02:01^_(CLG)_ The capacity of iIL-18_LMP2A_TCR-T cells to induce bystander immune cell activation upon recognition of autologous EBV^+^ B-LCL^A∗02:0^^1^_(CLG)_ was evaluated via (A and B) triple co-cultures containing effector, target and bystander cells as well as (C and D) chemotaxis assay. (iIL-18_)LMP2A_TCR-T cells were generated from human primary CD8^+^ T cells isolated from HLA-A∗02:01^+^ healthy donors using lentiviral transduction, while untransduced CD8^+^ T cells served as negative control (effector cells). EBV^+^ B-LCL^A∗^^02:01^ were generated using PBMCs isolated from the same donors, and EBV^+^ B-LCL^A^^∗^^02:01^_CLG_ were prepared by overnight loading with CLG (target cells). (A and B) CD3^+^ T cells were isolated from the same donors and labeled with CellTrace Violet (CTV), followed by co-culture with target and effector cells (1:1:1 ratio). Co-cultures of bystander and target cells without effector cells served as negative control. After 72 h, activation (CD25 and CD69 expression) of bystander CD8^+^ and CD4^+^ T cells was assessed via flow cytometry. Summarized graphs show the frequencies of CD25^+^CD69^+^ bystander CD8^+^ and CD4^+^ T cells in presence of (A) EBV^+^ B-LCL^A∗^^02:01^ or (B) EBV^+^ B-LCL^A∗^^02:01^_CLG_ (target cells) and effector cells as indicated. Data are shown as mean ± SD, and each symbol represents data from one independent donor (*n* = 9). (C and D) Chemotaxis potential of cell culture supernatant collected from effector cells as indicated exposed to (C) EBV^+^ B-LCL^A∗^^02:01^ or (D) EBV^+^ B-LCL^A∗^^02:01^_CLG_ was evaluated by live cell imaging. Summarizing graphs show quantification of migrating THP-1 cells over time. Data are shown as mean ± SD (*n* = 5). Statistical analysis was performed using two-way ANOVA and Sidak’s multiple comparisons test. ∗*p* ≤ 0.05; ∗∗*p* ≤ 0.01; ∗∗∗*p* ≤ 0.001; ∗∗∗∗*p* ≤ 0.0001.

Since IL-18 not only provides autocrine effects but also acts in a paracrine way,^33^ the effect of secreted IL-18 was first investigated on CD3^+^ bystander T cells (CellTrace violet (CTV)-labeled CD3^+^ T cells) in co-cultures with autologous target cells (EBV^+^ B-LCL^A^^∗^^02:01^_(CLG)_) in presence or absence of untransduced CD8^+^ T cells or (iIL-18_)LMP2A_TCR-T cells (Figure 4).

Analysis of activation amongst CD8^+^ and CD4^+^ bystander T cells revealed significantly increased frequencies of CD25^+^CD69^+^ cells amongst CD8^+^ bystander T cells in co-cultures with EBV^+^ B-LCL^A∗^^02:01^ and iIL-18_LMP2A_TCR-T cells but not LMP2A_TCR-T cells (Figure 4A). In presence of EBV^+^ B-LCL^A∗^^02:01^_CLG_ as target cells, activation of CD8^+^ bystander T cells was significantly increased in presence of both, LMP2A_TCR- and iIL-18_LMP2A-TCR-T cells when compared to untransduced CD8^+^ T cells or EBV^+^ B-LCL^A∗^^02:01^_CLG_ only (Figure 4B). Of note, iIL-18_LMP2A-TCR-T were significantly superior to LMP2A_TCR-T cells in activation of CD8^+^ bystander T cells in presence of EBV^+^ B-LCL^A∗^^02:01^_CLG_. Generally, no effects on CD4^+^ bystander T cells were observed under these experimental conditions (Figures 4A and 4B).

To elucidate the effects of IL-18 secreted by iIL-18_LMP2A_TCR-T cells on innate bystander cells in more detail, a chemotaxis assay was performed. To this end, cell culture supernatants from co-cultures containing EBV^+^ B-LCL^A∗^^02:01^_(CLG)_ and (iIL-18_)LMP2A_TCR-T cells were collected and used to investigate their potential to attract THP-1 cells. While supernatants from (iIL-18_)LMP2A_TCR-T cells exposed to EBV^+^ B-LCL^A∗^^02:01^ did not affect THP-1 cell migration (Figure 4C), significantly increased migration of THP-1 cells was observed in presence of supernatants from both, iIL-18_LMP2A_TCR-T and LMP2A_TCR-T cells exposed to EBV^+^ B-LCL^A∗^^02:01^_CLG_, when compared to supernatants from EBV^+^ B-LCL^A∗^^02:01^_CLG_ alone or in presence of untransduced CD8^+^ T cells (Figure 4D). Of note, supernatants obtained from iIL-18_LMP2A_TCR-T cells encountering EBV^+^ B-LCL^A∗^^02:01^_CLG_ induced significantly stronger migration when compared to LMP2A_TCR-T cells.

Overall, these data indicate that iIL-18 released by iIL-18_LMP2A_TCR-T cells upon target cell encounter has the potential to induce bystander T cell activation and recruit innate immune cells, enabling further control of the EBV^+^ tumor.

### iIL-18_LMP2A_TCR-T cells control growth of a 3D *in vitro* EBV^+^ multicellular tumor spheroid model

**Figure 5:**
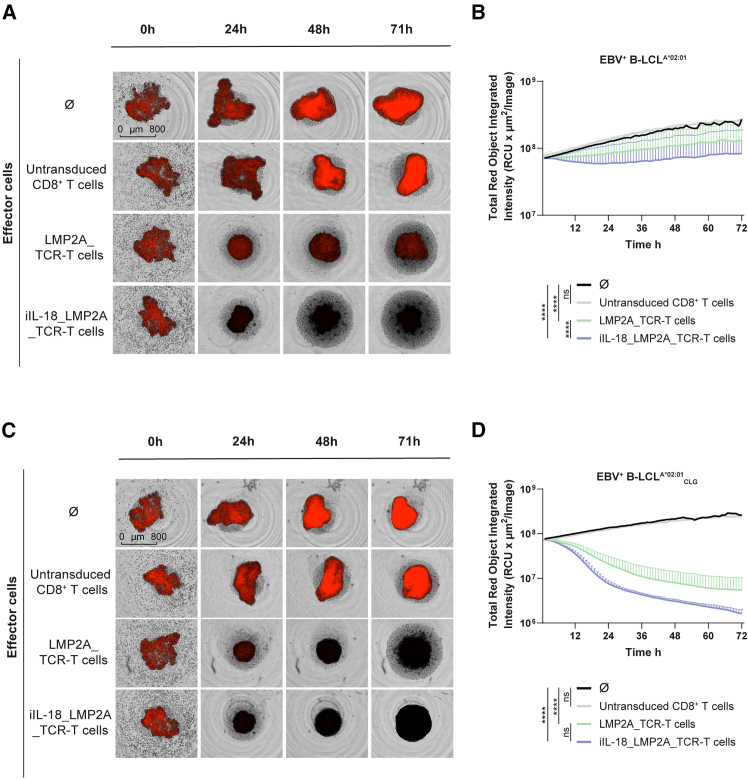
iIL-18_LMP2A_TCR-T cells reduce growth of mCherry-B-LCL^A∗^^02:01^_CLG_ multicellular tumor spheroids (MCTS) The specific recognition and elimination of allogeneic EBV^+^ B-LCL^A∗^^02:01^ by (iIL-18_)LMP2A_TCR-T cells in a challenging *in vivo*-like environment was evaluated using multicellular tumor spheroids (MCTS) and live cell imaging. For that, (iIL-18_)LMP2A_TCR-T cells were generated from human primary CD8^+^ T cells via lentiviral transduction. Untransduced CD8^+^ T cells served as negative control. EBV^+^ B-LCL^A∗^^02:01^ were generated using PBMCs isolated from HLA-A∗02:01^+^ healthy donors, followed by lentiviral transduction to express mCherry (mCherry-EBV^+^ B-LCL^A∗^^02:01^). mCherry-EBV^+^ B-LCL^A∗^^02:01^_CLG_ were prepared by overnight loading with the HLA-A∗02:01-restricted LMP2A-derived peptide CLGGLLTMV (CLG). MCTS were formed using mCherry-EBV^+^ B-LCL^A∗^^02:01^_(CLG)_ and HLA-I-deficient Bj-5ta fibroblast cells and exposed to (iIL-18_)LMP2A_TCR-T cells or untransduced CD8^+^ T cells. Growth of MCTS was evaluated using IncuCyte SX1 applying 4× objective lenses. Scale bars in images represent 800 μm. (A) Representative overlay of brightfield and red fluorescence images of EBV^+^ B-LCL^A∗^^02:01^ MCTS cultured in presence of effector cells as indicated at indicated time points. (B) Quantitative analysis of MCTS growth indicated by the Total Red Object Integrated Intensity (RCU x μm^2^/image) as mean + SD (*n* = 3). (C) Representative overlay of brightfield and red fluorescence images of EBV^+^ B-LCL^A∗^^02:01^_CLG_ MCTS cultured in presence of effector cells as indicated at indicated time points. (D) Quantitative analysis of MCTS growth indicated by the Total Red Object Integrated Intensity (RCU x μm^2^/image) as mean + SD (*n* = 3). Statistical analysis was performed using two-way ANOVA and Tukey’s multiple comparisons test. Results are shown for comparisons to MCTS only and comparison between LMP2A_TCR-T cells and iIL-18_LMP2A_TCR-T cells. ns not significant; ∗∗∗∗*p* ≤ 0.0001.

To examine functionality of (iIL-18_)LMP2A_TCR-T cells in a more complex environment that mimics the tumor, multicellular tumor spheroids (MCTSs) consisting of HLA-I-deficient BJ-5ta fibroblast cells and mCherry-EBV^+^ B-LCL^A∗^^02:01^_(CLG)_ were generated and co-cultured with either (iIL-18_)LMP2A_TCR-T cells or untransduced CD8^+^ T cells for 72 h (Figure 5). Live cell imaging showed that the growth of EBV^+^ B-LCL^A∗^^02:01^ within MCTSs was controlled by both (iIL-18_)LMP2A_TCR-T cells, while it remained unaffected by untransduced CD8^+^ T cells (Figures 5A and 5B). Notably, iIL-18_LMP2A_TCR-T cells were significantly superior in controlling MCTS growth when compared to LMP2A_TCR-T cells (Figure 5B). In MCTSs containing EBV^+^ B-LCL^A∗^^02:01^_CLG_, iIL-18_LMP2A_TCR-T cells and LMP2A_TCR-T cells significantly controlled MCTS growth over time (Figures 5C and 5D). While iIL-18_LMP2A_TCR-T cells appeared to be more potent compared to LMP2A_TCR-T cells, this difference was not statistically significant.

In conclusion, MCTS models for EBV^+^ PTLD demonstrated that (iIL-18_)LMP2A_TCR-T cells control growth of EBV^+^ B-LCL^A∗^^02:01^ cells in a challenging environment, with iIL-18_LMP2A_TCR-T cells showing significantly superior activity compared to LMP2A_TCR-T cells.

## Discussion

In immunocompromised individuals, uncontrolled proliferation of EBV-infected B cells due to the lack of a functional antiviral T cell immunity can lead to development of different malignancies. These include EBV^+^ PTLD, which is, along with other EBV^+^ tumors of latency types II/III, associated with the expression of the EBV-derived protein LMP2A. In this study, we successfully developed genetically engineered T cells that constitutively express a TCR targeting the LMP2A-derived HLA-A∗02:01-restricted epitope A∗02_LMP2A_CLG_ and were additionally equipped with an inducible cassette for IL-18 release to combat EBV^+^ PTLD and other EBV-associated malignancies. These iIL-18_LMP2A_TCR-T cells were able to eliminate EBV^+^ B-LCL^A∗^^02:01^, serving as *in vitro* EBV^+^ PTLD model, thereby marking a crucial advance in the development of effective immunotherapeutic approaches against EBV-associated malignancies, with low risk of on-target/off-tumor toxicity due to the exclusive presence of the targeted antigen on EBV-infected cells.

LMP2A is expressed in EBV latency stages II/III and its expression is associated with the development of PTLD as well as various malignancies and carcinomas.^28^^,^^29^ Previous immunotherapeutic approaches using naturally occurring LMP2A-specific T cells have been facing challenges, partly due to their low frequencies, thus requiring long expansion protocols.^34^ While TCR-engineered T cells harbor the potential to overcome such hurdles, these require knowledge of immunodominant epitopes and corresponding specific TCR sequences. The LMP2A-derived peptide CLG presented in context of HLA-A∗02:01 (A∗02_LMP2A_CLG_) presents a promising and clinically relevant target for immunotherapy due to the crucial role of LMP2A for tumor survival, as well as the high prevalence of HLA-A∗02:01 in the European population.^31^^,^^35^ In this study, an LMP2A_CLG_-specific TCR was used that has recently been identified by TCR sequencing of T cells expanded in presence of the CLG peptide.^32^ When expressed in primary T cells, the LMP2A_CLG_-specific TCR was found to produce IFN-γ upon recognition of EBV^+^ B-LCLs, which are characterized by an EBV latency III expression profile.^32^^,^^36^^,^^37^ It was further demonstrated that LMP2A undergoes effective processing in EBV^+^ B-LCLs, resulting in presentation of LMP2A-derived peptides as well as concentration-dependent binding of A∗02_LMP2A_CLG_ by TCR-like monoclonal antibodies.^37^ Using avidity measurements, binding of the LMP2A_TCR to EBV^+^ B-LCL^A∗^^02:01^ was confirmed, suggesting presence of A∗02_LMP2A_CLG_ on the surface of EBV^+^ B-LCL^A∗^^02:01^ as well as specificity of the LMP2A_TCR. However, for clinical application of iIL-18_LMP2A_TCR-T cells, further investigation of the safety profile including any potential off-target effects is required.

EBV has evolved several immune evasion strategies, including downregulation of components of the antigen processing machinery and interference with peptide loading.^38^^,^^39^ Depending on disease entity and the microenvironment (e.g., upregulation of HLA class I by interferons, downregulation of HLA class I by IL-10),^40^ HLA class I expression can be expected to vary both in a patient-associated fashion as well as in the context of different malignancies. In Hodgkin lymphoma, loss or downregulation of HLA-I is frequently observed in Reed-Sternberg cells, representing a key mechanism of immune escape.^41^ In contrast, HLA-I expression in PTLD and EBV^+^ DLBCL is often retained at detectable levels, allowing for recognition by EBV-specific cytotoxic T cells.^42^ These differences have direct implications for TCR-based immunotherapy, as the therapeutic efficacy relies on sufficient HLA-I surface expression combined with presentation of suitable EBV-derived peptides. Therefore, although EBV^+^ Hodgkin lymphoma as well as DLBCL are characterized by latency II/III and therefore by expression of LMP2A, the therapeutic potential of iIL-18_LMP2A_TCR-T cells for treatment of EBV^+^ Hodgkin lymphoma remains to be investigated.

In patients post-SOT, the risk of graft rejection, such as it has been reported after treatment with anti-CD19 CAR-T cells,^23^ is to be critically evaluated. In case of anti-CD19 CAR-T cells, B cells are depleted systemically including those in the circulation. This is expected to be different in case of (iIL-18_)LMP2A_TCR-T cells, since these only recognize EBV-infected but not healthy B cells, thereby preventing on-target-off tumor effects. Furthermore, this targeted approach reduces the number of target cells, which could have a positive effect on minimizing side effects, as it is well known that severe side effects are more likely to occur, particularly in cases of high tumor burden. Therefore, activation of (iIL-18_)LMP2A_TCR-T cells is expected to be more localized compared to anti-CD19 CAR-T cells. This critical safety aspect will require rigorous evaluation in preclinical models and clinical trials. The impact of immunosuppressive treatment needs to be taken into consideration. Immunosuppression is a key clinical factor in PTLD, as drugs like calcineurin inhibitors and glucocorticoids can limit TCR-T cell efficacy. Inducible IL-18 may help counteract these effects and strategies such as generating T cells resistant to immunosuppressants have already been established to preserve and maintain engineered T cell functions.^43^^,^^44^^,^^45^

In 2021, Zhang et al. demonstrated specificity and cytolytic activity of LMP2A-specific TCR-engineered T cells against lymphoblastoid cell lines that overexpress LMP2A.^46^ While overcoming the bottleneck of low frequencies of naturally occurring LMP2A-specific T cells, the study also highlighted several challenges, including limited persistence of the TCR-engineered T cells. CAR- or TCR-engineered T cells further equipped for constitutive or inducible cytokine release, such as IL-12 or IL-18, have been developed to modulate the TME and thereby enhance their therapeutic efficacy.^26^^,^^47^^,^^48^ Intravenous infusion of recombinant IL-18 in advanced cancer patients was found to be well tolerated and associated with transient lymphocyte and monocyte activation,^49^ suggesting IL-18 as a safe and efficient approach to improve T cell functionality. In this study, inducible IL-18 release by LMP2A_TCR-T cells resulted in slightly higher activation upon target recognition, indicating a direct impact of IL-18 on the TCR-engineered T cells. Importantly, this led to an overall superior and more sustained cytotoxic capacity, which resulted in efficient elimination of EBV^+^ B-LCL^A∗^^02:01^. This is in line with previous reports, showing that IL-18-releasing TCR-T cells targeting a melanoma antigen increased CD8^+^ T cell infiltration into tumors, reduced tumor burden, and prolonged survival in a mouse model.^47^ Similarly, 4^th^ generation GD2-CAR-T cells armored with IL-18 have demonstrated enhanced efficacy and cytotoxicity and are currently being tested in neuroblastoma patients (EU CT 2022-501725-21-00).^50^^,^^51^ Alongside increased cytotoxicity, slightly higher levels of IFN-γ, TNF-α, and perforin were secreted by iIL-18_LMP2A_TCR-T cells when compared to LMP2A_TCR-T cells, indicating a type 1-like phenotype, which is in line with the reported induction of T helper 1 (Th1) responses by IL-18 in CD4^+^ T cells.^52^^,^^53^ Further, slightly higher levels of IL-2 were observed, which might act not only in an autocrine but also in a paracrine way. While TNF-α and IFN-γ can have direct antitumor effects, IL-2 supports the proliferation and differentiation of effector cells, thereby potentially contributing to improved persistence potential.^54^ IL-12-releasing 4^th^ generation CAR-T cells have shown potential to modulate the TME, however, IL-12 has been associated with severe toxicities and higher levels of T cell exhaustion, especially in systems with constitutive IL-12 expression.^48^^,^^55^ Similarly, IL-12-related toxicities have been observed in TCR-engineered T cells equipped with IL-12.^47^^,^^56^ Therefore, while equipment of LMP2A_TCR-T cells with inducible IL-12 may be an alternative approach for the treatment of EBV-associated malignancies, this approach requires detailed safety assessment.

Besides the autocrine effect of IL-18 on CAR- or TCR-engineered T cells, IL-18 was shown to activate innate bystander cells such as NK cells.^53^ Further, increased recruitment of monocytes by 4^th^ generation CAR-T cells targeting GD2 was observed compared to 2^nd^ generation CAR-T cells.^50^ The enhanced functionality and cytotoxicity of iIL-18_LMP2A_TCR-T cells observed in this study is at least in part due to autocrine IL-18 signaling, which was especially apparent in settings with limited antigen presentation. This points to a possible niche for IL-18 - enhancing effector function in settings of suboptimal peptide presentation, which closely mirrors the expected *in vivo* situation. Accordingly, our data suggest that iIL-18_LMP2A_TCR-T cells, via release of iIL-18, have the potential to activate and recruit bystander cells that can contribute to clearance of EBV-infected cells. Moreover, while in the *in vitro* bystander assay CD4^+^ bystander T cells were not found to be activated in presence of iIL-18_LMP2A_TCR-T cells, which was in contrast to their CD8^+^ counterparts, this may well be different *in vivo*. The potential importance of CD4^+^ T cell responses needs to be elucidated in future investigations. Of note, bystander activation was found to be independent of EBV serostatus of the T cell donor (data not shown), indicating antigen-independent mechanisms. This is in line with previous reports about activation of bystander T cells occurring independent of recognition of the cognate antigen.^57^^,^^58^^,^^59^^,^^60^ Taken together, IL-18 immunomodulatory effects such as recruitment and activation of innate and adaptive immune cells, induction of proinflammatory chemokines, and potential upregulation of MHC class I expression have the potential to contribute to tumor control. Whether recruitment and activation of innate as well as adaptive immune cells via IL-18 translate into enhanced antitumor effects remain to be investigated.

Immunotherapeutic approaches targeting LMP2A were so far limited by low persistence due to a terminally differentiated state.^46^^,^^61^ T cells can become functionally exhausted after prolonged antigen exposure within the TME and face significant barriers to infiltration, such as dense stroma and abnormal vasculature that impede T cell access to tumors.^62^^,^^63^ In this study, iIL-18_LMP2A_TCR-T cells exhibited significantly enhanced cytotoxic activity not only in 2D cultures but also in complex 3D *in vitro* models of EBV^+^ tumors (EBV^+^ B-LCL-MCTS). In the context of numerous tumors, cancer-associated fibroblasts (CAFs) are recognized as the predominant constituents of the TME, exerting crucial roles in facilitating physical barriers around tumors, fostering cancer cell proliferation, inducing immunosuppression to impede anti-tumor immune responses, and promoting resistance to therapeutic interventions.^64^ MCTS containing not only the EBV^+^ target cells but also fibroblasts therefore serve as invaluable tools that mimic the intricate molecular signaling, intercellular communication, and architectural barriers found *in vivo*. iIL-18_LMP2A_TCR-T cells not only efficiently infiltrated EBV^+^ B-LCL-MCTS but also reduced tumor growth over an extended period. Notably, LMP2A_TCR-T cells showed limited efficacy in the same setting, suggesting that iIL-18_LMP2A_TCR-T cells maintain robust functionality within the physical challenges of TME, highlighting their potential as a therapeutic strategy for targeting EBV-associated tumors.

### Conclusion

This study demonstrates the successful optimization of a T cell therapy approach using a TCR that specifically targets the clinically relevant A∗02_LMP2A_CLG_ epitope, which is expressed on malignant cells in EBV-associated lymphomas. An inducible cassette for IL-18, leading to locally restricted release of IL-18 upon recognition of the A∗02_LMP2A_CLG_ epitope, was incorporated to enhance T cell functionality, remodulate the immunosuppressive TME and attract bystander immune cells. iIL-18_LMP2A_TCR-T cells were superior to LMP2A_TCR-T cells, which was reflected in their significantly enhanced cytotoxic capacity toward EBV^+^ B-LCL^A∗^^02:01^ cells naturally processing intracellular EBV-derived proteins, presenting the A∗02_LMP2A_CLG_ epitope and thereby serving as *in vitro* PTLD model. Thus, by engineering patient-derived T cells to express the iIL-18_LMP2A_TCR, this approach represents a promising immunotherapeutic strategy to treat EBV-associated malignancies with a low risk of on-target/off-tumor toxicity. Our data present the significant anti-cancer potential of iIL-18_LMP2A_TCR-T cells, providing the groundwork for investigating the potential of iIL-18_LMP2A_TCR- and further TCR-T cell-based therapies for treatment of EBV-associated and other malignancies.

## Materials and methods

### Human sample material

Residual blood samples from platelet apheresis disposable kits, used for routine platelet collection, were obtained from EBV-seropositive and EBV-seronegative healthy donors at the Hannover Medical School (MHH) Institute of Transfusion Medicine and Transplant Engineering. All donors provided informed consent, and the study was approved by the Ethics Committee of MHH (ethical number: 3639–2017, 2744–2015). Donors’ EBV serostatus was pre-determined using a commercially available IgG western blot assay, as previously described.^65^^,^^66^ Peripheral blood mononuclear cells (PBMCs) were isolated via density gradient centrifugation using Lymphosep (C.C. Pro GmbH, Oberdorla, Germany). Possible background effects due to the presence of endogenous EBV-specific T cells are taken into account using untransduced CD8^+^ T cells as control.

### Cell lines and culture media

Cell lines and culture media used in this study are listed in Table 1. Cell numbers were determined using trypan blue exclusion and Neubauer chamber or a CellCountess device (Thermo Fisher Scientific, Waltham, MA, USA). EBV-immortalized B lymphoblastoid cell lines (EBV^+^ B-LCLs) were generated from PBMCs of HLA-A∗02:01^+^ healthy donors using established protocols.^68^ In brief, PBMCs were resuspended at a concentration of 2 × 10^6^/mL in transformation medium (Table 1). The cells were seeded into T25 flasks containing transformation media (TPP, Trasadingen, Switzerland) and infected with the EBV strain B95-8 at 37°C. After 10 days transformation media was replaced by culture media and cells were split once or twice a week. EBV-infected cells were monitored microscopically for the formation of rosette-like B-LCL clusters. Experiments were performed using EBV^+^ B-LCLs after at least four weeks of culture. Only EBV^+^ B-LCLs with baseline viability of above 30% were used for all experiments. To generate SPI-801^A∗^^02:01^ cells, SPI-801 cells were lentivirally transduced to express HLA-A∗02:01. HLA expression by SPI-801^A∗^^02:01^ and EBV^+^ B-LCL^A∗^^02:01^ was confirmed by flow cytometry using anti-HLA-ABC PE (clone W6/32) or anti-HLA-A2 FITC (clone BB7.2) antibodies (BioLegend). HLA-I knockout of Bj-5ta cells was performed using CRISPR-Cas9 as previously described.^69^

**Table 1:** Cell lines and culture media

Cells/cell line	Cell type	Supplier	Medium
HEK293T (ACC-635)	Derivative of human embryonal kidney cell line HEK, highly transfectable	DSMZ, Germany	DMEM 10% FBS (v/v) 2 mM L-glutamine
B-LCL	EBV-transformed B-lymphoblastic cell line	–	*Transformation medium* RPMI 1640 10% FBS (v/v) 200 ng/mL cyclosporin A *Culture medium* RPMI 1640 10% FBS (v/v) 2 mM L-glutamine 1% (v/v) Penicillin/Streptomycin
Jurkat (ACC-282)	T cell leukemia	DSMZ, Germany	RPMI 1640 10% FBS (v/v) 2 mM L-glutamine 1% (v/v) Penicillin/Streptomycin
JE6-1 transduced with reporter plasmids	Jurkat-derivative	Kindly provided by Prof. Steinberger^67^	RPMI 1640 10% FBS (v/v) 2 mM L-glutamine 1% (v/v) Penicillin/Streptomycin
SPI-801 (ACC-86) *HLA-A∗02:01-transduced*	Chronic myeloid leukemia in blast crisis	DSMZ, Germany	RPMI 1640 10% FBS (v/v) 2 mM L-glutamine 1% (v/v) Penicillin/Streptomycin
Bj-5ta (CRL-4001) *with HLA-I knockout*	Human foreskin fibroblast	ATCC	*4 parts* DMEM (high 4.5 g/L glucose) 4 mM L-glutamine *1 part* Medium 199 10% FBS
Primary T cells	T cell	–	Complete T cell (CTL) medium TexMACS 3% (v/v) human AB serum 1% (v/v) Penicillin/Streptomycin
THP-1	Monocyte	DSMZ, Germany	RPMI 1640 10% FBS (v/v) 2 mM L-glutamine

RPMI 1640 (PAN-Biotech GmbH), DMEM Medium (Gibco, Thermo Fisher Scientific, Waltham, MA, USA), TexMACS (Miltenyi Biotec), Medium 199 (Gibco, Thermo Fisher Scientific), Fetal bovine serum (FBS, PromoCell GmbH, Heidelberg, Germany), human AB serum (C.C. Pro GmbH), L-glutamine (Gibco, Thermo Fisher Scientific), cyclosporine (Novartis, Nuremberg, Germany), Penicillin/Streptomycin (C.C. Pro GmbH).

For selected co-cultures, target cells (SPI-801^A∗^^02:01^/EBV^+^ B-LCL^A∗^^02:01^) were labeled with CellTrace violet proliferation dye (CTV, Life Technologies, Thermo Fisher Scientific, Carlsbad, CA, USA) in serum-free RPMI 1640 (PAN-Biotech GmbH, Aidenbach, Germany) according to the manufacturer’s instructions. For peptide loading, 5 × 10^5^ target cells were seeded in a 24 well plate and incubated with 10 μg/mL (if not stated otherwise) of LMP2A-dervied peptide CLGGLLTMV (CLG) (peptides & elephants GmbH, Hennigsdorf, Germany) overnight in serum-free TexMACS medium (Miltenyi Biotec, Bergisch Gladbach, Germany).

### Generation of LMP2A_TCR constructs

The (iIL-18_)LMP2A_TCR constructs were designed by using the previously described CLG3A10a2 TCR sequence.^32^ To generate the iIL-18-secreting iIL-18_LMP2A_TCR and inducible enhanced green fluorescent protein (iEGFP)-expressing LMP2A_TCR constructs, the previously described “all-in-one” vectors containing either an NFAT-driven EGFP or IL-18 (matured cytokine without pro-peptide) expression cassette, respectively, and a constitutive LMP2A_TCR expression cassette were used.^50^

### Generation and titration of lentiviral particles

iIL-18_LMP2A_TCR and LMP2A_TCR lentiviral particles were produced in HEK293T cells using the calcium phosphate method. In brief, a plasmid containing the sequence of interest, along with the packaging plasmid pcDNA3.HIV-1.GP.4 × CTE (encoding gag and pol),^70^ the envelope plasmid VSVg-encoding pMD.G (encoding env),^71^ and the pRSV-Rev plasmid (kindly provided by T. Hope, Northwestern University, Chicago, IL, USA, encoding rev), were transfected into HEK293T cells. Viral particle-containing supernatants were collected, filtered, and concentrated by ultracentrifugation. The resulting pellet was resuspended in 1 mL of DMEM supplemented with 20 mM HEPES (both Gibco, Thermo Fisher Scientific) and stored at −80°C. Viral titers were determined by transducing Jurkat cells in the presence of 5 μg/μL Polybrene (EMD Millipore Corporation, Burlington, MA, USA). Transduction efficiency was assessed 48 h post-transduction through staining with mTCR-β antibody (clone H57-597-PE; BioLegend, San Diego, CA, USA), followed by flow cytometric analysis (BD FACSCanto II, BD Biosciences, Heidelberg, Germany).

### Reporter assay to determine (iIL-18_)LMP2A TCR signaling

A previously documented reporter cell line derived from the Jurkat JE6-1 T cell line was transduced with lentiviral particles (multiplicity of infection [MOI] = 1–3) using 5 μg/mL Polybrene (EMD Millipore Corporation) and spinoculation.^67^ Target cell lines (SPI-801^A∗^^02:01^_(CLG)_/EBV^+^ B-LCL^A∗^^02:01^_(CLG)_) were labeled with CTV and peptide loaded as described above. TPR assays were set as previously described.^27^ Briefly, after peptide loading, 1 × 10^5^ CTV-labeled target cells were co-cultured with 1 × 10^5^ transduced JE6-1 reporter cells in a 1:1 ratio in 200 μL CTL medium. Transduced JE6-1 reporter cells stimulated with Dynabeads Human T activator CD3/CD28 (Gibco, Thermo Fisher Scientific) served as positive control. Signaling of iIL-18_LMP2A_TCR and LMP2A_TCR was determined after 48 h through the measurement of enhanced cyan fluorescent protein (eCFP) expression as an indicator of NF-kB activation by flow cytometry (BD FACSCanto II, BD Biosciences).

### Generation of primary iIL-18_LMP2A_TCR-T cells and LMP2A_TCR-T cells

Untouched CD8^+^ T cells were isolated from healthy donor PBMCs using the CD8^+^ T cell isolation kit (Miltenyi Biotec) and magnetic sorting (MACS) according to the manufacturer’s instructions. For transduction, the isolated CD8^+^ T cells were activated using Dynabeads Human T activator CD3/CD28 (Thermo Fisher Scientific) at a cell to bead ratio of 1:1 in CTL medium supplemented with 12.5 ng/mL IL-7 and IL-15 (both from PeproTech, Inc., Cranbury, NJ, USA). After one day, T cells were transduced with previously described lentiviral particles (MOI 1–3) via spinoculation in the presence of 5 μg/mL Polybrene. CD3/CD28 beads were removed on day 2 and cells were split based on their growth approximately every other day. On day 8/9, (iIL-18_)LMP2A_TCR-T cells were magnetically enriched using the murine constant domain (mTCR) by using biotinylated anti-mTCR-ß antibody (clone H57-597, BioLegend) and anti-biotin microbeads (Miltenyi Biotec). Enriched T cells were expanded until day 12–14. Throughout the entire process, untransduced CD8^+^ T cells were treated identically (with exception of enrichment via mTCR) and served as control for background activity due alloreactivity (in allogeneic experimental setups) or presence of endogenous EBV-specific T cells (in case of EBV-seropositive donors) in all experiments.

### Characterization of (iIL-18_)LMP2A_TCR-T cells via flow cytometry

(iIL-18_)LMP2A_TCR-T cells were phenotypically and functionally analyzed throughout their generation and upon encounter of target cells using multicolor flow cytometry (BD FACSCanto II, BD Biosciences). For determining T cell activation, a panel including anti-CD3-PerCP (clone SK7), anti-CD8-Brilliant Violet (BV) 510 (clone SK7), anti-CD25-PE-Cyanine7 (PE-Cy7) (clone S20019D), anti-CD69-BV605 (clone FN50), and anti-CD137-APC (clone 4B4-1) was used (all BioLegend). For determining T cell phenotype, this panel was extended to include anti-CD45RO-APC-Cyanine7 (clone UCHL1), anti-CD95-BV 421 (clone DX2), anti-CCR7-AF 700 (clone G043H7) and anti-mTCR-β-PE (clone H57-597). A panel consisting of anti-CD3-PerCP (clone SK7), anti-CD4-AF 700 (clone SK7), anti-CD8-BV 510 (clone SK7), anti-TIM-3-APC (clone F38-2E2), and anti-LAG 3-BV 605 (clone EH12.2H7) was used for evaluation of T cell exhaustion (all BioLegend). The cells were stained for 20 min at room temperature in the dark and washed with PBS. For analysis of their cytotoxic capacity, 7-aminoactinomycin D (7-AAD; BD Biosciences) staining was employed to identify dead CTV^+^ target cells. Data were analyzed using FlowJo v.10.8.1 (BD Biosciences). Gates were set based on the forward scatter versus side scatter properties of leukocytes. At least 10,000 events were acquired in the leukocyte gate.

### Evaluation of iIL-18_LMP2A_TCR and LMP2A_TCR-T cell avidity

Microfluidic chips were treated with 1 M KOH prior to coating with poly-L-lysine (Sigma-Aldrich, St. Louis, Missouri, USA). Chips were dried in a 37°C incubator and unbound poly-L-lysine was removed. EBV^+^ B-LCL^A∗^^02:01^_CLG_ were seeded into coated chips at a density of 1.5–2.0 × 10^8^ cells/mL to achieve a confluent surface and allowed to adhere for 1 h prior to measurement on the z-Movi Cell Avidity Analyzer (LUMICKS, Amsterdam, Netherlands). Subsequently, chips were washed with fresh CTL medium to remove unbound cells and allow formation of a target cell monolayer and confirmed via transmitted light microscopy. Autologous T cells, generated from the same donor as the EBV^+^ B-LCL^A∗^^02:01^_(CLG)_, were labeled with CellTrace Far Red (CTR) Proliferation Kit (Invitrogen, Thermo Fisher Scientific) for 10 min at 37°C according to the manufacturer’s instructions. CTR-labeled T cells were adjusted to a density of 1 × 10^7^ cells/mL, added to the chips and incubated for 5 min prior to acoustic force application. Force ramped up from 0 to 1,000 pN over 150 s. Detachment of single T cells from the target cell monolayer was analyzed using Oceon software (LUMICKS) according to manufacturer’s recommendations.

### Evaluation of iIL-18_LMP2A_TCR-T-cell functionality

To assess functionality of (iIL-18_)LMP2A_TCR-T cells, they were cultured in presence of CTV-labeled target cells (SPI-801^(A^^∗^^02:01)^_(CLG)_/EBV^+^ B-LCL^A^^∗^^02:01^_CLG_), prepared as described above, in an effector to target (E:T) ratio of 1:1 for 48 h. Activation and cytotoxic potential of the (iIL-18_)LMP2A_TCR-T cells was evaluated using flow cytometry as described above. Data shown in Figure 2 were performed in autologous or allogeneic settings, with a mean HLA class I match of 2/6 (range 0/6–5/6) between (iIL-18_)LMP2A_TCR-T cells and EBV^+^ B-LCL^A∗^^02:01^_(CLG)_ (allogeneic setting). Possible effects due to alloreactivity are taken into account using untransduced CD8^+^ T cells as control.

### Cytokine profiling by multiplex analysis

Cell culture supernatants were collected from co-cultures of T cells and their respective target cells after 48 h. Cytokine secretion levels were determined using customized a LEGENDplex Multi-Analyte Flow Assay (BioLegend) according to the manufacturer’s instructions. Samples were measured by flow cytometry and analyzed with respect to the concentration of human IL-2, IL-18, granzyme B, granzyme A, granulysin, perforin, IFN-γ and TNF-α. Data were analyzed with LEGENDplex v.8.0 software (BioLegend).

### Time-lapse imaging for real-time evaluation of killing of EBV^+^ B-LCLs by (iIL-18_)LMP2A_TCR-T cells *in vitro*

For live-cell imaging, B-LCL^A∗^^02:01^_CLG_ cells were seeded at a density of 50,000 cells/well in 96-well plates. The next day, (iIL-18_)LMP2A_TCR-T cells were added at an E:T ratio of 1:1. Cell death was assessed by Propodium Iodide staining (PI) (Miltenyi Biotec) added at a final concentration of 100 ng/mL. Images were acquired every hour using the Incucyte SX1 Live-Cell Analysis System (Sartorius, Göttingen, Germany) GUI Software (version 2022B Rev2) at 37°C in 5% CO_2_. This experiment was performed in autologous (*n* = 1) or allogeneic settings (*n* = 2), with an HLA class I match between (iIL-18_)LMP2A_TCR-T cells and EBV^+^ B-LCL^A∗^^02:01^_(CLG)_ of 1/6 (allogeneic setting). Possible effects due to alloreactivity are taken into account using untransduced CD8^+^ T cells as control.

### Triple-co-culture for assessment of bystander T cell activation

(iIL-18_)LMP2A_TCR_T-cells were generated as described above. CD3^+^ T cells (bystander T cells) were isolated using Pan T cell Isolation Kit (Miltenyi Biotec) and MACS according to the manufacturer’s instructions. Isolated CD3^+^ T cells were stored at −80°C until use. For triple-co-cultures, the CD3^+^ T cells were thawed, rested in CTL media overnight and labeled with CTV the following day. 5 × 10^4^ bystander T cells and 5 × 10^4^ target cells (EBV^+^ B-LCL^A∗^^02:01^_(CLG)_) were cultured in presence or absence of 5 × 10^4^ effector cells (untransduced CD8^+^ T cells or (iIL-18_)LMP2A_TCR-T cells). After 72 h, cells were harvested, stained with anti-CD3-AF 700 (clone HIT3a), anti-CD4-BV 510 (clone RPA-T4), anti-CD8-APC (clone SK1), anti-CD25-APC-Cyanine7 (clone BC96), anti-CD69-BV605 (clone FN50), anti-CD19-PE-Cyanine7 (clone HIB19), and anti-CD20-PE-Cyanine7 (clone 2H7). Samples were acquired at BD FACSCanto II (BD Biosciences). Cell culture supernatants of co-cultures without bystander CD3^+^ T cells were collected and stored at −20°C until use in chemotaxis assays.

### Chemotaxis assay for evaluation of innate immune cell migration by iIL-18

For evaluation of chemotaxis potential of collected supernatants, chemotaxis assays with THP-1 cells were performed using the Incucyte SX1 Live-Cell Analysis System (Sartorius). ClearView plate coating with Matrigel (Corning) was done following the manufacturer’s instructions. Briefly, both sides of the ClearView plate membrane were coated with 50 μg/mL Matrigel diluted in RPMI with 0.5% BSA by adding 20 μL to the insert wells and 150 μL to reservoir wells. After placing the insert into the reservoir, the plate was incubated at 37°C for 30 min, and an additional 30 min at room temperature. Reservoir coating was aspirated and replaced with 200 μL of PBS, and prior to cell seeding, insert coating was removed. 5 × 10^3^ THP-1 cells in RPMI, 0.5% FCS were added to each well of the insert plate and allowed to settle for 45–60 min at room temperature. Supernatants collected as described above were diluted 1:2 with RPMI, 0.5% FCS and added to the reservoir plate. The insert plate with THP-1 cells was transferred to the reservoir plate containing supernatants and warmed to 37°C for 15 min. The plate was transferred into the Incucyte SX1 Live-Cell Analysis System (Sartorius) and images were captured every hour using 10× objective lenses and the Chemotaxis (Top/Bot) scan type. The area covered by migrated THP-1 cells on the bottom of the membrane was analyzed using the IncuCyte 2023A Rev2 software (Sartorius) and normalized to the corresponding initial top values.

### Functional evaluation of iIL-18_LMP2A_TCR-T cells using B-LCL multicellular tumor spheroid

mCherry-EBV^+^ B-LCLs were generated through transduction of EBV^+^ B-LCLs with lentivirus pRRL.PPT.SF.mCherry.pre (kindly provided by Prof. Schambach/Prof. Baum/Prof. Morgan, Hannover Medical School, Germany) at an MOI of 1 via spinoculation in the presence of 5 μg/mL Polybrene and FACS-sorted using mCherry expression. Next, the MCTS were formed by incubation of HLA-I-deficient BJ-5ta and mCherry-EBV^+^ B-LCLs at a 2:1 ratio. For that, a total of 5,000 cells per wells were plated into 96-well Ultra-low Attachment PrimeSurface Cell Culture U-bottom Plates (Sbio, Hudson, USA) in 200 μL CTL medium. For peptide loading, 10 μg/mL of LMP2A-dervied peptide CLGGLLTMV (CLG) (peptides & elephants GmbH) were added. After 3 days, 100 μL medium was removed and (iIL-18_)LMP2A_TCR-T cells were added in 100 μL CTL medium. The HLA class I match between (iIL-18_)LMP2A_TCR-T cells and EBV^+^ B-LCL^A∗^^02:01^_(CLG)_ ranged between 0/6 (*n* = 1) and 1/6 (*n* = 2). Possible effects due to alloreactivity are taken into account using untransduced CD8^+^ T cells as control. The cells were cultured at 37°C in 5% CO_2_ and analyzed with the Incucyte SX1 Live-Cell Analysis System (Sartorius). During 48 h of incubation, cell viability and spheroid growth was assessed via measurement of the red fluorescence (mCherry) signal. Images were captured every hour using 4× objective lenses and analyzed using the IncuCyte 2022B Rev2 software (Sartorius).

### Statistical analysis

Statistical analysis was performed with GraphPad Prism V10 using two-way ANOVA, 1-way ANOVA, or Kruskal-Wallis test, followed by uncorrected Dunn's test, Sidaks multiple comparison test or mixed-effects analysis with Tukey’s test, as indicated in the respective figure legends. ns: not significant; ∗*p* ≤ 0.05; ∗∗*p* ≤ 0.01; ∗∗∗*p* ≤ 0.001; ∗∗∗∗*p* ≤ 0.0001.

## Data and code availability

The datasets and protocols used and/or analyzed during the current study are available from the corresponding author (eiz-vesper.britta@mh-hannover.de) on reasonable request and upon completion of a material transfer agreement.

## Acknowledgments

P.M. was supported by the Hannover Biomedical Research School (HBRS). This project was in part supported by the Ellen-Schmidt-Program of Hannover Medical School (to A.B.), by the nextGENERATION Medical Scientist Program funded by 10.13039/501100003042Else Kröner-Fresenius Foundation (2022_EKMK.13; to A.C.D. and A.B.), by the 10.13039/501100001659Deutsche Forschungsgemeinschaft (DFG.; SFB900, FOR2830; TRR338 LetsImmun, Subproject A02 to M.H., Subproject A05 to T.N.), by the 10.13039/100009139German Center for Infection Research (DZIF; TTU07.838_00, TI 07.003_007 MD program [to S.S.])), Deutsche Krebshilfe e.V. (70115705 to L.H.; 70114707 and 70115200 to M.H.) and the Bavarian Center for Cancer Research (Bayerisches Zentrum für Krebsforschung, BZKF, Leuchtturm Immuntherapie, Projekt Präklinische Entwicklung). The authors wish to thank Sarina Lukis, Elvira Schulde, and Kai Loβner for technical support, Prof. Dr. med. Axel Schambach/Prof. Dr. Christopher Baum/Prof. Dr. Michael Morgan (MHH, Hannover, Germany) for providing the mCherry lentivirus and Dr. Peter Steinberger (10.13039/501100005788Medical University of Vienna, Austria) for providing the JE6-1 reporter cell line. The authors would further like to thank Dr. Manuel Vicente for support in chemotaxis assays (Institute of Clinical Biochemistry, MHH, Hannover, Germany), Dr. Murielle Verboom for HLA typing (Institute of Transfusion Medicine and Transplant Engineering, MHH, Hannover, Germany), and Dr. Matthias Ballmaier for cell sorting (Central Research Facility Cell Sorting, MHH, Hannover, Germany).

## Author contributions

Conceptualization, A.B., P.M., A.C.D., and B.E.-V.; methodology, A.B., P.M., F.F., A.C.D., S.T.-Z., P.R., and P.S.; software, A.B. and P.M.; validation, A.B., P.M., and B.E.-V.; formal analysis, A.B., P.M., A.C.D., P.S., and B.E.-V.; investigation, A.B., P.M., F.F., A.C.D., and B.E.-V.; resources, M.F.L.C., A.H., H.A., R.B., T.N., M.H., A.S., L.H., B.M.-K., and B.E.-V.; data curation, A.B., P.M., and B.E.-V.; writing – original draft preparation, A.B. and P.M.; writing – review and editing, all authors; visualization, A.B. and P.M.; supervision, A.B. and B.E.-V.; project administration, A.B. and B.E.-V.; funding acquisition, B.E.-V.

## Declaration of interests

The authors declare that the research was conducted in the absence of any commercial or financial relationships that could be construed as a potential conflict of interest. A.S. and H.A. have an active patent for “All-in one vector for car and therapeutic effector molecule” (EP3986428A1). L.H. served on advisory committees for Bristol Myers Squibb, Gilead, Johnson & Johnson, Pierre-Fabre, and Sanofi and received travel support from Amgen, Gilead, and Johnson & Johnson, all unrelated to this study.

## Supplemental information

Document S1. Figures S1–S3

Document S2. Article plus supplemental information

## References

[1] Martorelli D., Muraro E., Merlo A., Turrini R., Faè D.A., Rosato A., Dolcetti R. (2012). Exploiting the interplay between innate and adaptive immunity to improve immunotherapeutic strategies for Epstein-Barr-virus-driven disorders. Clin. Dev. Immunol..

[2] Kang M.S., Kieff E. (2015). Epstein-Barr virus latent genes. Exp. Mol. Med..

[3] El-Sharkawy A., Al Zaidan L., Malki A. (2018). Epstein-Barr Virus-Associated Malignancies: Roles of Viral Oncoproteins in Carcinogenesis. Front. Oncol..

[4] Thorley-Lawson D.A. (2015). EBV Persistence--Introducing the Virus. Curr. Top. Microbiol. Immunol..

[5] Shannon-Lowe C., Rickinson A. (2019). The Global Landscape of EBV-Associated Tumors. Front. Oncol..

[6] Schober T., Framke T., Kreipe H., Schulz T.F., Grohennig A., Hussein K., Baumann U., Pape L., Schubert S., Wingen A.M. (2013). Characteristics of early and late PTLD development in pediatric solid organ transplant recipients. Transplantation.

[7] Allen U.D., Preiksaitis J.K., Practice A.S.T.I.D.C.o. (2019). Post-transplant lymphoproliferative disorders, Epstein-Barr virus infection, and disease in solid organ transplantation: Guidelines from the American Society of Transplantation Infectious Diseases Community of Practice. Clin. Transplant..

[8] Dierickx D., Tousseyn T., Gheysens O. (2015). How I treat posttransplant lymphoproliferative disorders. Blood.

[9] O’Reilly R.J., Prockop S., Oved J.H. (2024). Virus-specific T-cells from third party or transplant donors for treatment of EBV lymphoproliferative diseases arising post hematopoietic cell or solid organ transplantation. Front. Immunol..

[10] Chaganti S., Barlev A., Caillard S., Choquet S., Cwynarski K., Friedetzky A., González-Barca E., Sadetsky N., Schneeberger S., Thirumalai D. (2023). Expert Consensus on the Characteristics of Patients with Epstein-Barr Virus-Positive Post-Transplant Lymphoproliferative Disease (EBV(+) PTLD) for Whom Standard-Dose Chemotherapy May be Inappropriate: A Modified Delphi Study. Adv. Ther..

[11] Al Hamed R., Bazarbachi A.H., Mohty M. (2020). Epstein-Barr virus-related post-transplant lymphoproliferative disease (EBV-PTLD) in the setting of allogeneic stem cell transplantation: a comprehensive review from pathogenesis to forthcoming treatment modalities. Bone Marrow Transplant..

[12] Amengual J.E., Pro B. (2023). How I treat posttransplant lymphoproliferative disorder. Blood.

[13] Vase M.Ø., Maksten E.F., Bendix K., Hamilton-Dutoit S., Andersen C., Møller M.B., Sørensen S.S., Jespersen B., Kampmann J., Søndergård E. (2015). Occurrence and prognostic relevance of CD30 expression in post-transplant lymphoproliferative disorders. Leuk. Lymphoma.

[14] Prockop S., Doubrovina E., Suser S., Heller G., Barker J., Dahi P., Perales M.A., Papadopoulos E., Sauter C., Castro-Malaspina H. (2020). Off-the-shelf EBV-specific T cell immunotherapy for rituximab-refractory EBV-associated lymphoma following transplantation. J. Clin. Investig..

[15] Jiang W., Clancy L.E., Avdic S., Sutrave G., Street J., Simms R., McGuire H.M., Patrick E., Chan A.S., McCaughan G. (2022). Third-party CMV- and EBV-specific T-cells for first viral reactivation after allogeneic stem cell transplant. Blood Adv..

[16] Pfeiffer T., Tzannou I., Wu M., Ramos C., Sasa G., Martinez C., Lulla P., Krance R.A., Scherer L., Ruderfer D. (2023). Posoleucel, an Allogeneic, Off-the-Shelf Multivirus-Specific T-Cell Therapy, for the Treatment of Refractory Viral Infections in the Post-HCT Setting. Clin. Cancer Res..

[17] Bonifacius A., Lamottke B., Tischer-Zimmermann S., Schultze-Florey R., Goudeva L., Heuft H.G., Arseniev L., Beier R., Beutel G., Cario G. (2023). Patient-tailored adoptive immunotherapy with EBV-specific T cells from related and unrelated donors. J. Clin. Investig..

[18] Haque T., Wilkie G.M., Jones M.M., Higgins C.D., Urquhart G., Wingate P., Burns D., McAulay K., Turner M., Bellamy C. (2007). Allogeneic cytotoxic T-cell therapy for EBV-positive posttransplantation lymphoproliferative disease: results of a phase 2 multicenter clinical trial. Blood.

[19] Mahadeo K.M., Baiocchi R., Beitinjaneh A., Chaganti S., Choquet S., Dierickx D., Dinavahi R., Duan X., Gamelin L., Ghobadi A. (2024). Tabelecleucel for allogeneic haematopoietic stem-cell or solid organ transplant recipients with Epstein-Barr virus-positive post-transplant lymphoproliferative disease after failure of rituximab or rituximab and chemotherapy (ALLELE): a phase 3, multicentre, open-label trial. Lancet Oncol..

[20] Binnewies M., Roberts E.W., Kersten K., Chan V., Fearon D.F., Merad M., Coussens L.M., Gabrilovich D.I., Ostrand-Rosenberg S., Hedrick C.C. (2018). Understanding the tumor immune microenvironment (TIME) for effective therapy. Nat. Med..

[21] Wang Q., Shao X., Zhang Y., Zhu M., Wang F.X.C., Mu J., Li J., Yao H., Chen K. (2023). Role of tumor microenvironment in cancer progression and therapeutic strategy. Cancer Med..

[22] Giraldo N.A., Sanchez-Salas R., Peske J.D., Vano Y., Becht E., Petitprez F., Validire P., Ingels A., Cathelineau X., Fridman W.H., Sautès-Fridman C. (2019). The clinical role of the TME in solid cancer. Br. J. Cancer.

[23] McKenna M., Epperla N., Ghobadi A., Liu J., Lazaryan A., Ibrahim U., Jacobson C.A., Naik S.G., Nastoupil L., Chowdhury S.M. (2023). Real-world evidence of the safety and survival with CD19 CAR-T cell therapy for relapsed/refractory solid organ transplant-related PTLD. Br. J. Haematol..

[24] Abbas F., Kossi M.E., Shaheen I.S., Sharma A., Halawa A. (2020). Post-transplantation lymphoproliferative disorders: Current concepts and future therapeutic approaches. World J. Transplant..

[25] Braun T., Pruene A., Darguzyte M., Vom Stein A.F., Nguyen P.H., Wagner D.L., Kath J., Roig-Merino A., Heuser M., Riehm L.L. (2023). Non-viral TRAC-knocked-in CD19(KI)CAR-T and gp350(KI)CAR-T cells tested against Burkitt lymphomas with type 1 or 2 EBV infection: In vivo cellular dynamics and potency. Front. Immunol..

[26] Chmielewski M., Abken H. (2017). CAR T Cells Releasing IL-18 Convert to T-Bet(high) FoxO1(low) Effectors that Exhibit Augmented Activity against Advanced Solid Tumors. Cell Rep..

[27] Dragon A.C., Zimmermann K., Nerreter T., Sandfort D., Lahrberg J., Klöß S., Kloth C., Mangare C., Bonifacius A., Tischer-Zimmermann S. (2020). CAR-T cells and TRUCKs that recognize an EBNA-3C-derived epitope presented on HLA-B∗35 control Epstein-Barr virus-associated lymphoproliferation. J. Immunother. Cancer.

[28] Cheever M.A., Allison J.P., Ferris A.S., Finn O.J., Hastings B.M., Hecht T.T., Mellman I., Prindiville S.A., Viner J.L., Weiner L.M., Matrisian L.M. (2009). The prioritization of cancer antigens: a national cancer institute pilot project for the acceleration of translational research. Clin. Cancer Res..

[29] Busson P., Edwards R.H., Tursz T., Raab-Traub N. (1995). Sequence polymorphism in the Epstein-Barr virus latent membrane protein (LMP)-2 gene. J. Gen. Virol..

[30] Lee S.P., Thomas W.A., Murray R.J., Khanim F., Kaur S., Young L.S., Rowe M., Kurilla M., Rickinson A.B. (1993). HLA A2.1-restricted cytotoxic T cells recognizing a range of Epstein-Barr virus isolates through a defined epitope in latent membrane protein LMP2. J. Virol..

[31] Hurley C.K., Kempenich J., Wadsworth K., Sauter J., Hofmann J.A., Schefzyk D., Schmidt A.H., Galarza P., Cardozo M.B.R., Dudkiewicz M. (2020). Common, intermediate and well-documented HLA alleles in world populations: CIWD version 3.0.0. HLA.

[32] Lammoglia Cobo M.F., Welters C., Rosenberger L., Leisegang M., Dietze K., Pircher C., Penter L., Gary R., Bullinger L., Takvorian A. (2022). Rapid single-cell identification of Epstein-Barr virus-specific T-cell receptors for cellular therapy. Cytotherapy.

[33] Hull C.M., Larcombe-Young D., Mazza R., George M., Davies D.M., Schurich A., Maher J. (2024). Granzyme B-activated IL18 potentiates alphabeta and gammadelta CAR T cell immunotherapy in a tumor-dependent manner. Mol. Ther..

[34] Bollard C.M., Gottschalk S., Torrano V., Diouf O., Ku S., Hazrat Y., Carrum G., Ramos C., Fayad L., Shpall E.J. (2014). Sustained complete responses in patients with lymphoma receiving autologous cytotoxic T lymphocytes targeting Epstein-Barr virus latent membrane proteins. J. Clin. Oncol..

[35] Sausen D.G., Poirier M.C., Spiers L.M., Smith E.N. (2023). Mechanisms of T cell evasion by Epstein-Barr virus and implications for tumor survival. Front. Immunol..

[36] Bollard C.M., Rooney C.M., Heslop H.E. (2012). T-cell therapy in the treatment of post-transplant lymphoproliferative disease. Nat. Rev. Clin. Oncol..

[37] Lai J., Tan W.J., Too C.T., Choo J.A.L., Wong L.H., Mustafa F.B., Srinivasan N., Lim A.P.C., Zhong Y., Gascoigne N.R.J. (2016). Targeting Epstein-Barr virus-transformed B lymphoblastoid cells using antibodies with T-cell receptor-like specificities. Blood.

[38] Hislop A.D., Ressing M.E., van Leeuwen D., Pudney V.A., Horst D., Koppers-Lalic D., Croft N.P., Neefjes J.J., Rickinson A.B., Wiertz E.J.H.J. (2007). A CD8+ T cell immune evasion protein specific to Epstein-Barr virus and its close relatives in Old World primates. J. Exp. Med..

[39] Horst D., van Leeuwen D., Croft N.P., Garstka M.A., Hislop A.D., Kremmer E., Rickinson A.B., Wiertz E.J.H.J., Ressing M.E. (2009). Specific targeting of the EBV lytic phase protein BNLF2a to the transporter associated with antigen processing results in impairment of HLA class I-restricted antigen presentation. J. Immunol..

[40] Hazini A., Fisher K., Seymour L. (2021). Deregulation of HLA-I in cancer and its central importance for immunotherapy. J. Immunother. Cancer.

[41] Müller-Meinhard B., Seifert N., Grund J., Reinke S., Yalcin F., Kaul H., Borchmann S., von Tresckow B., Borchmann P., Plütschow A. (2024). Human leukocyte antigen (HLA) class I expression on Hodgkin-Reed-Sternberg cells is an EBV-independent major determinant of microenvironment composition in classic Hodgkin lymphoma. HemaSphere.

[42] Nguyen-Van D., Keane C., Han E., Jones K., Nourse J.P., Vari F., Ross N., Crooks P., Ramuz O., Green M. (2011). Epstein-Barr virus-positive diffuse large B-cell lymphoma of the elderly expresses EBNA3A with conserved CD8 T-cell epitopes. Am J Blood Res.

[43] Amini L., Wagner D.L., Rössler U., Zarrinrad G., Wagner L.F., Vollmer T., Wendering D.J., Kornak U., Volk H.D., Reinke P., Schmueck-Henneresse M. (2021). CRISPR-Cas9-Edited Tacrolimus-Resistant Antiviral T Cells for Advanced Adoptive Immunotherapy in Transplant Recipients. Mol. Ther..

[44] Kaeuferle T., Deisenberger L., Jablonowski L., Stief T.A., Blaeschke F., Willier S., Feuchtinger T. (2020). CRISPR-Cas9-Mediated Glucocorticoid Resistance in Virus-Specific T Cells for Adoptive T Cell Therapy Posttransplantation. Mol. Ther..

[45] Dragon A.C., Bonifacius A., Lienenklaus S., Verboom M., Gerhards J.P., Ius F., Hinze C., Hudecek M., Figueiredo C., Blasczyk R., Eiz-Vesper B. (2025). Depletion of alloreactive B cells by drug-resistant chimeric alloantigen receptor T cells to prevent transplant rejection. Mol. Ther..

[46] Zhang C., Tan Q., Li S., Shen L., Zhang J., Liu Y., Yang W., Lu Z. (2021). Induction of EBV latent membrane protein-2A (LMP2A)-specific T cells and construction of individualized TCR-engineered T cells for EBV-associated malignancies. J. Immunother. Cancer.

[47] Kunert A., Chmielewski M., Wijers R., Berrevoets C., Abken H., Debets R. (2017). Intra-tumoral production of IL18, but not IL12, by TCR-engineered T cells is non-toxic and counteracts immune evasion of solid tumors. Oncoimmunology.

[48] Pietrobon V., Todd L.A., Goswami A., Stefanson O., Yang Z., Marincola F. (2021). Improving CAR T-Cell Persistence. Int. J. Mol. Sci..

[49] Robertson M.J., Mier J.W., Logan T., Atkins M., Koon H., Koch K.M., Kathman S., Pandite L.N., Oei C., Kirby L.C. (2006). Clinical and biological effects of recombinant human interleukin-18 administered by intravenous infusion to patients with advanced cancer. Clin. Cancer Res..

[50] Zimmermann K., Kuehle J., Dragon A.C., Galla M., Kloth C., Rudek L.S., Sandalcioglu I.E., Neyazi B., Moritz T., Meyer J. (2020). Design and Characterization of an “All-in-One” Lentiviral Vector System Combining Constitutive Anti-G(D2) CAR Expression and Inducible Cytokines. Cancers (Basel).

[51] Fischer-Riepe L., Kailayangiri S., Zimmermann K., Pfeifer R., Aigner M., Altvater B., Kretschmann S., Völkl S., Hartley J., Dreger C. (2024). Preclinical Development of CAR T Cells with Antigen-Inducible IL18 Enforcement to Treat GD2-Positive Solid Cancers. Clin. Cancer Res..

[52] Nakanishi K., Yoshimoto T., Tsutsui H., Okamura H. (2001). Interleukin-18 regulates both Th1 and Th2 responses. Annu. Rev. Immunol..

[53] Ihim S.A., Abubakar S.D., Zian Z., Sasaki T., Saffarioun M., Maleknia S., Azizi G. (2022). Interleukin-18 cytokine in immunity, inflammation, and autoimmunity: Biological role in induction, regulation, and treatment. Front. Immunol..

[54] Ross S.H., Cantrell D.A. (2018). Signaling and Function of Interleukin-2 in T Lymphocytes. Annu. Rev. Immunol..

[55] Koneru M., O’Cearbhaill R., Pendharkar S., Spriggs D.R., Brentjens R.J. (2015). A phase I clinical trial of adoptive T cell therapy using IL-12 secreting MUC-16(ecto) directed chimeric antigen receptors for recurrent ovarian cancer. J. Transl. Med..

[56] Drakes D.J., Rafiq S., Purdon T.J., Lopez A.V., Chandran S.S., Klebanoff C.A., Brentjens R.J. (2020). Optimization of T-cell Receptor-Modified T Cells for Cancer Therapy. Cancer Immunol. Res..

[57] Cornelis R., Shulman Z. (2023). Bystander activation of tissue-resident memory CD4 T cells: Getting by with a little help from unfamiliar T-cell friends. Eur. J. Immunol..

[58] Kim T.S., Shin E.C. (2019). The activation of bystander CD8(+) T cells and their roles in viral infection. Exp. Mol. Med..

[59] Lee H.G., Cho M.J., Choi J.M. (2020). Bystander CD4(+) T cells: crossroads between innate and adaptive immunity. Exp. Mol. Med..

[60] Yosri M., Dokhan M., Aboagye E., Al Moussawy M., Abdelsamed H.A. (2024). Mechanisms governing bystander activation of T cells. Front. Immunol..

[61] Huang J., Fogg M., Wirth L.J., Daley H., Ritz J., Posner M.R., Wang F.C., Lorch J.H. (2017). Epstein-Barr virus-specific adoptive immunotherapy for recurrent, metastatic nasopharyngeal carcinoma. Cancer.

[62] Kanakry J.A., Ambinder R.F. (2013). EBV-related lymphomas: new approaches to treatment. Curr. Treat. Options Oncol..

[63] Heslop H.E., Sharma S., Rooney C.M. (2021). Adoptive T-Cell Therapy for Epstein-Barr Virus-Related Lymphomas. J. Clin. Oncol..

[64] Peltier A., Seban R.D., Buvat I., Bidard F.C., Mechta-Grigoriou F. (2022). Fibroblast heterogeneity in solid tumors: From single cell analysis to whole-body imaging. Semin. Cancer Biol..

[65] Heinemann N.C., Tischer-Zimmermann S., Wittke T.C., Eigendorf J., Kerling A., Framke T., Melk A., Heuft H.G., Blasczyk R., Maecker-Kolhoff B., Eiz-Vesper B. (2020). High-intensity interval training in allogeneic adoptive T-cell immunotherapy - a big HIT?. J. Transl. Med..

[66] Bieling M., Tischer S., Kalinke U., Blasczyk R., Buus S., Maecker-Kolhoff B., Eiz-Vesper B. (2018). Personalized adoptive immunotherapy for patients with EBV-associated tumors and complications: Evaluation of novel naturally processed and presented EBV-derived T-cell epitopes. Oncotarget.

[67] Jutz S., Leitner J., Schmetterer K., Doel-Perez I., Majdic O., Grabmeier-Pfistershammer K., Paster W., Huppa J.B., Steinberger P. (2016). Assessment of costimulation and coinhibition in a triple parameter T cell reporter line: Simultaneous measurement of NF-kappaB, NFAT and AP-1. J. Immunol. Methods.

[68] Hui-Yuen J., McAllister S., Koganti S., Hill E., Bhaduri-McIntosh S. (2011). Establishment of Epstein-Barr virus growth-transformed lymphoblastoid cell lines. J. Vis. Exp..

[69] de Waard A.A., Verkerk T., Jongsma M.L.M., Hoefakker K., Sethumadhavan S., Gerke C., Bliss S., Kong X., Janssen G.M.C., de Ru A.H. (2021). PAKC: A novel panel of HLA class I antigen presentation machinery knockout cells from the same genetic origin. Eur. J. Immunol..

[70] Schambach A., Bohne J., Chandra S., Will E., Margison G.P., Williams D.A., Baum C. (2006). Equal potency of gammaretroviral and lentiviral SIN vectors for expression of O6-methylguanine-DNA methyltransferase in hematopoietic cells. Mol. Ther..

[71] Yang Y., Vanin E.F., Whitt M.A., Fornerod M., Zwart R., Schneiderman R.D., Grosveld G., Nienhuis A.W. (1995). Inducible, high-level production of infectious murine leukemia retroviral vector particles pseudotyped with vesicular stomatitis virus G envelope protein. Hum. Gene Ther..

