# Supplementary material for: Next-generation LMP2A-targeting TCR-recombinant T cells with inducible IL-18 expression to treat EBV-associated malignancies
Source: Mol Ther Oncol. 2026 Jun 11;34(3):201265. doi: 10.1016/j.omton.2026.201265 (PMC13334400; doi:10.1016/j.omton.2026.201265)
Supplement: Document S1. Figures S1–S3 [file mmc1.pdf]

OMTON, Volume *34*

Supplemental information

Next-generation LMP2A-targeting TCR-recombinant T cells with inducible IL-18 expression to treat EBV-associated malignancies

Agnes Bonifacius, Philip Mausberg, Friederike Floegel, Anna Christina Dragon, Sabine Tischer-Zimmermann, Sven Stoll, Pegah Rahmati, Peter Spieler, María Fernanda Lammoglia Cobo, Anne Halenius, Hinrich Abken, Rainer Blasczyk, Thomas Nerreter, Michael Hudecek, Axel Schambach, Leo Hansmann, Britta Maecker-Kolhoff, and Britta Eiz-Vesper

Supplemental Information

Figure S1

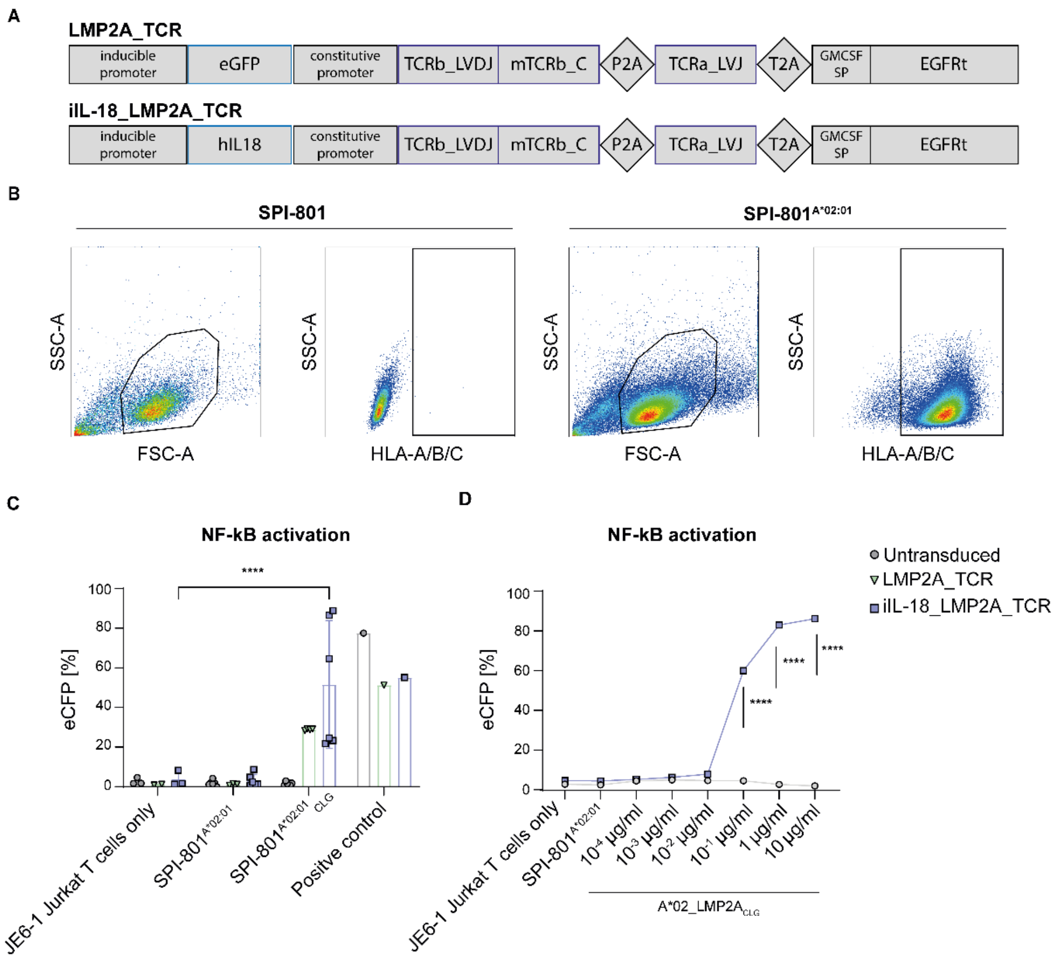

Figure S1: (iIL-18_)LMP2A_TCR-expressing JE6-1 reporter cells are specifically activated upon recognition of the A*02_LMP2A$_{CLG}$ epitope on SPI-801$^{A*02:01}$$_{CLG}$ cells.

(A) Schematic representation of lentiviral constructs for generation of LMP2A_TCR- and iIL-18_LMP2A_TCR-T cells. **(B)** As proof-of-concept target cells, SPI-801 cells expressing HLA-A*02:01 (SPI-801$^{A*02:01}$) were generated by lentiviral transduction. SPI-801$^{A*02:01}$$_{CLG}$ were prepared by overnight loading with the HLA-A*02:01-restricted LMP2A-derived peptide CLGGLLTMV (CLG). (iIL-18_)LMP2A-TCR-expressing JE6-1 reporter cells were generated by lentiviral transduction. Representative pseudocolour dot plots show expression of HLA-A*02:01 by SPI-801$^{A*02:0}$ but not SPI-801, indicated by staining with a pan-HLA-I antibody. **(C)** (iIL-18_)LMP2A-TCR-expressing JE6-1 reporter cells were cultured in presence or absence of CellTrace Violet (CTV)-labeled SPI-801$^{A*02:01}$$_{CLG}$ in a ratio of 1:1. Corresponding reporter cells stimulated with anti-CD3/CD28 beads were used as positive control. After 48 h, NF-κB activation, indicated by expression of enhanced cyan fluorescent protein (eCFP), was evaluated via flow cytometry (n=1-6). **(D)** iIL-18_LMP2A-TCR-expressing JE6-1 reporter cells were cultured in presence or absence of CellTrace Violet (CTV)-labeled SPI-801$^{A*02:01}$$_{CLG}$

loaded with indicated concentrations of CLG in a ratio of 1:1 for 48 h, followed by flow cytometric analysis of NF-κB activation, indicated by expression of eCFP (n=2-3). **(C-D)** Data are shown as mean±SD, **(C)** each symbol represents data from one technical replicate. Statistical analysis was performed using Two Way ANOVA and Dunnett's multiple comparisons test. ****$p \leq 0.0001$.

Figure S2

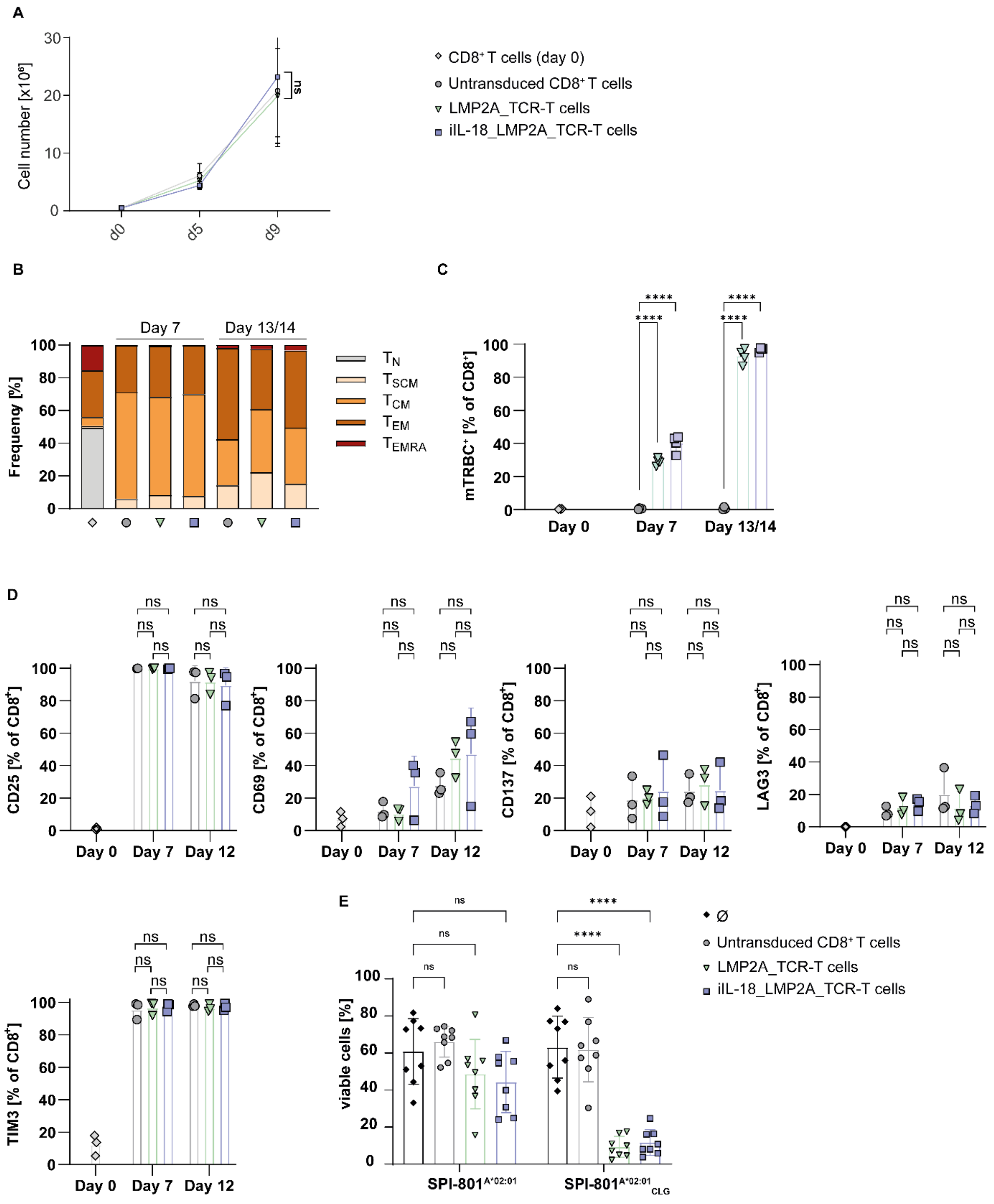

Figure S2: Successful generation of (iIL-18_)LMP2A_TCR-T cells which specifically eliminate SPI-801$^{A*02:01}$$_{CLG}$ cells.

(iIL-18_)LMP2A_TCR-T cells were generated from human primary CD8^{+} T cells via lentiviral transduction. Untransduced CD8^{+} T cells served as negative control. **(A)** Cell numbers during generation, shown as mean±SD (n=8). **(B)** Memory phenotype and **(C)** frequency of mTRBC^{+} cells during generation (n=4). **(D)** The activation/exhaustion phenotype of (iIL-18_)LMP2A_TCR-T cells during generation was evaluated by multicolour flow cytometry. Summarizing graphs show the frequencies of LAG3^{+}, TIM3^{+}, CD69^{+}, CD137^{+} and CD25^{+} cells among CD8^{+} T cells. **(B-D)** Data are

shown as **(B)** mean or **(C-D)** mean±SD, each symbol represents data from one donor. Statistical analysis was performed for each day using Two Way ANOVA and Tukey's multiple comparison. **(E)** The cytotoxic capacity of (iIL-18_)LMP2A_TCR-T cells towards SPI-801$^{A*02:01}{}_{CLG}$ cells was evaluated using flow cytometry. For that, CellTrace Violet (CTV)-labeled SPI-801$^{A*02:01}{}_{(CLG)}$ cells were subjected to (iIL-18)_LMP2A_TCR-T cells (1:1 ratio) for 48 h, followed by quantification of viable target cells (7-AAD^{-} CTV^{+}). Target cells cultured in absence of T cells served as control for baseline viability. Data are shown as mean±SD, each symbol represents data from one donor. Statistical analysis was performed using Two Way ANOVA and Dunnett's multiple comparisons test. ns not significant; ****p≤ 0.0001.

Figure S3

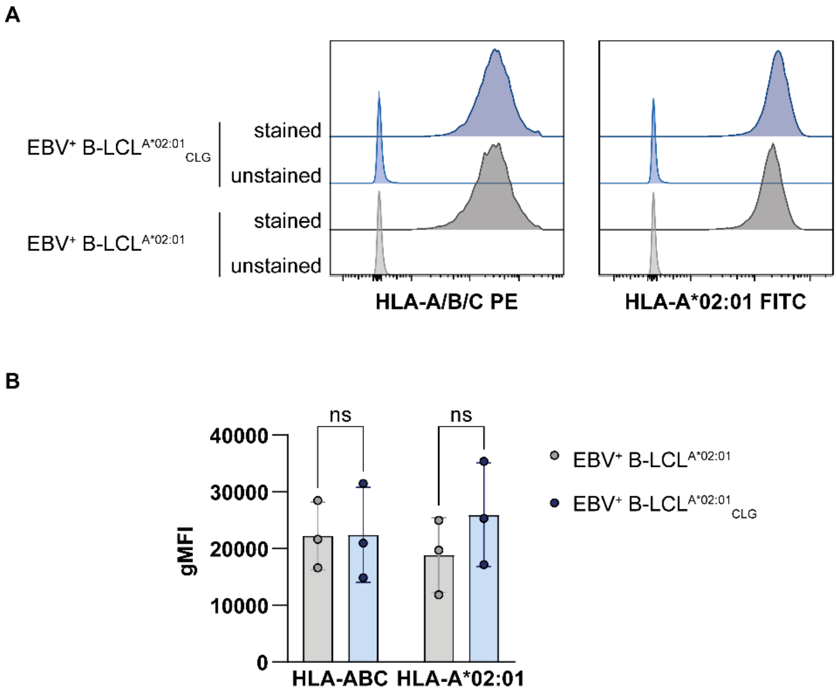

Figure S3: HLA expression by EBV$^+$ B-LCLs.
EBV$^+$ B-LCL$^{A*02:01}{}_{(CLG)}$ were assessed for expression of HLA-A/B/C and HLA-A2 by flow cytometry. **(A)** Representative histograms and **(B)** summarized bar graphs. Each dot represents data from one cell line (donor). Statistical analysis was performed using Two Way ANOVA and Sidak's multiple comparisons test. ns not significant.
